# Nanoenhanced‐Cuproptosis Results From the Synergy of Calcium Overload and GSH Depletion with the Increasing of Intracellular Ca/Mn/Cu Ions

**DOI:** 10.1002/advs.202412067

**Published:** 2025-02-10

**Authors:** Shiwei Liu, Wennan Yan, Wenyue Zhang, Ji Zhang, Ziyi Li, Yingshu Guo, Hong‐Yuan Chen, Jing‐Juan Xu

**Affiliations:** ^1^ School of Chemistry and Chemical Engineering Qilu University of Technology (Shandong Academy of Sciences) Jinan 250353 China; ^2^ State Key Laboratory of Analytical Chemistry for Life Science School of Chemistry and Chemical Engineering Nanjing University Nanjing 210023 China

**Keywords:** calcium overload, cuproptosis, Fenton‐like reaction of manganese, GSH depletion

## Abstract

Cuproptosis is a newly discovered copper‐dependent form of cell death. Intracellular glutathione (GSH) acts as a copper chelator to inhibit cuproptosis, so the reduction of GSH concentration is conducive to enhancing the cuproptosis of cells. In order to reduce GSH content and interfere with mitochondrial metabolism, a strategy based on calcium overload and GSH depletion to enhance cuproptosis is proposed in this study. Containing manganese (Mn) and copper (Cu) elements, CaCO_3_ nanoparticles (NPs) are modified with MCF‐7 cell aptamer (CaCO_3_/Mn/Cu@lip‐Apt). When entering the cell, CaCO_3_/Mn/Cu@lip‐Apt decomposed and released Mn* (Mn^2+^/Mn^3+^/Mn^4+^), Cu^2+^ and Ca^2+^. The high valence Mn ion in Mn* can effectively consume GSH to produce Mn^2+^ which catalyzed H_2_O_2_ to produce reactive oxygen species (ROS), while reducing the GSH concentration. The production of ROS promoted the influx of exogenous Ca^2+^. The large accumulation of Ca^2+^ led to intracellular calcium overload, resulting in mitochondrial dysfunction and metabolism disorders. The depletion of GSH promoted the accumulation of Cu^2+^, which in turn triggered cuproptosis. This strategy showed excellent antitumor effects and provided a new way to study disease treatment.

## Graphical abstract

In this study, CaCO_3_/Mn/Cu@lip‐Apt release Mn*, Cu^2+^, and Ca^2+^ in cells. The high valence Mn ion in Mn* can effectively consume GSH, allowing more Cu^2+^ to participate in cuproptosis. This situation will induce the oligomerization of DLAT. The large accumulation of Ca^2+^ leads to intracellular calcium overload, resulting in metabolism disorders. Finally, the proposed strategy enhanced cuproptosis.

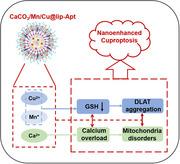

## 1 Introduction

Cu is a vital trace element involved in many biological processes.^[^ ^1^ ^]^ During the normal physiological process, Cu is always maintained at a low level to achieve Cu homeostasis. However, when copper ions (Cu^2+^) concentration is excessive, Cu homeostasis is disrupted and leads to cell death.^[^ ^2^ ^]^ Cuproptosis is a novel copper‐dependent death route that has been discovered recently.^[^ ^3^ ^]^ Distincting from all other known processes, such as apoptosis, necroptosis, pyroptosis, and ferroptosis, it depends on the excessive accumulation of Cu in cells.^[^ ^4^ ^]^ The direct interaction of lipoylated components involved in the tricarboxylic acid cycle with Cu^2+^ caused the aggregation of lipoylated proteins, following degradation of iron‐sulfur cluster proteins (such as ferredoxin 1, FDX‐1) and accumulation of toxic proteins (such as dihydroacylamide S‐acetyltransferase, DLAT), ultimately leading to cell death.^[^ ^3^ ^]^ By taking advantage of the unique properties of cuproptosis, this process may facilitate research into Cu in the treatment of tumors.^[^ ^5^ ^]^ However, cells are able to efficiently eliminate excess Cu^2+^ through Cu^2+^ transporters, thus preventing the accumulation of Cu^2+^ in the cells.^[^ ^6^ ^]^ This mechanism makes it more difficult for Cu^2+^ to accumulate within cells.^[^ ^7^ ^]^ Many small molecule compounds have been used to deliver copper (Cu^+^ or Cu^2+^) into cells, such as dithiocarbamates, bis(thiosemicarbazone) ligands, elecromol, 8‐hydroxyquinoline etc.^[^ ^8^ ^]^ However, one of the limitations of therapeutic approaches based on cuproptosis is the lack of specific targeted delivery of copper. This situation may trigger increased toxicity because Cu accumulates indiscriminately in healthy cells or tissues.^[^ ^9^ ^]^ Therefore, selectively delivering Cu to target sites and preventing its premature release are expected to improve the efficacy of treatments while avoiding potential risks of toxicity.^[^ ^10^ ^]^

To overcome this challenge, researchers have turned their attention to nanoparticles (NPs).^[^ ^11^ ^]^ NPs possess the ability to control the release of their contents based on the tumor microenvironment, such as temperature, pH levels, and redox potential.^[^ ^12^ ^]^ Nonetheless, the unmodified NPs lack the ability to differentiate between different cell types. The surface modification method using nucleic acid aptamers can significantly enhance the targeting of NPs to specific cells and reduce toxicity to healthy tissues.^[^ ^13^ ^]^

In recent years, calcium‐based NPs including calcium carbonate (CaCO_3_) NPs,^[^ ^14^ ^]^ calcium peroxide NPs,^[^ ^15^ ^]^ and calcium phosphate NPs^[^ ^16^ ^]^ have been developed for calcium overload‐mediated cancer treatment. It has been reported that as a key intracellular molecule, excessive Ca^2+^ can interfere with mitochondrial metabolism, downregulate mitochondrial membrane potential, and reduce the production of adenosine triphosphate (ATP), thereby inducing concentration‐dependent apoptosis.^[^ ^17^ ^]^ At the same time, as an ideal initiator of intracellular calcium overload, calcium‐based NPs can provide excess Ca^2+^, which leads to respiratory chain electron leakage and further enhances the generation of intracellular ROS.^[^ ^18^ ^]^ On the one hand, the accumulation of ROS within cells can cause excessive oxidative stress to cell death. On the other hand, a large amount of GSH in the cell reduces the accumulation of ROS.^[^ ^19^ ^]^ In cuproptosis, the GSH depletion can increase the oligomerization of the DLAT and sensitize cells to cuproptosis. To balance oxidative stress, GSH is upregulated, which severely hinders cuproptosis in diseased cells. In other words, GSH depletion can increase the efficiency of cuproptosis, and GSH upregulation blocks the cuproptosis pathway.^[^ ^3^ ^]^ At this point, the role of Mn becomes prominent, which is essential for regulating the activities of a variety of enzymes with different functions.^[^ ^20^ ^]^ High valent Mn can consume GSH generating Mn^2+^. Mn^2+^ catalyzes H_2_O_2_ to generate ROS.^[^ ^21^ ^]^ ROS could promote the influx of Ca^2+^ through stimulating the transient receptor potential A1 (TRPA1) protein, which is overexpressed in tumor cells, thereby enhancing the calcium overloading effect.^[^ ^22^ ^]^ Therefore, nanotechnology for the sustained release of Ca^2+^ and the production of ROS is beneficial for enhancing anti‐tumor effects by causing mitochondrial dysfunction.^[^ ^23^ ^]^

Considering the current attention of nanomedicine research on the anti‐cancer strategy of cuproptosis, we developed a CaCO_3_/Mn/Cu NP, which was loaded Mn^*^(Mn^2+^/ Mn^3+^/ Mn^4+^)and Cu^2+^. Then a layer of lipid and a nucleic acid aptamer targeting breast cancer cells (MCF‐7 cells) were coated on its surface successively, which is called CaCO_3_/Mn/Cu@lip‐Apt. This design enabled CaCO_3_/Mn/Cu@lip‐Apt to be efficiently taken up by MCF‐7 cells, resulting in increased intracellular calcium concentrations, mitochondrial dysfunction, and susceptibility to cuproptosis. At the same time, the higher‐valent Mn* released by NPs can consume GSH, which reduces the consumption of ROS (for Ca^2+^ influx) and allows more Cu^2+^ to participate in cuproptosis. Therefore, under the synergistic effect of calcium overload and GSH consumption, our strategy significantly improved the cuproptosis effect and provided new ideas for breast cancer treatment.

## 2 Results and Discussion

### 2.1 Therapeutic Mechanism of CaCO_3_/Mn/Cu@lip‐Apt

**Figure 1:**
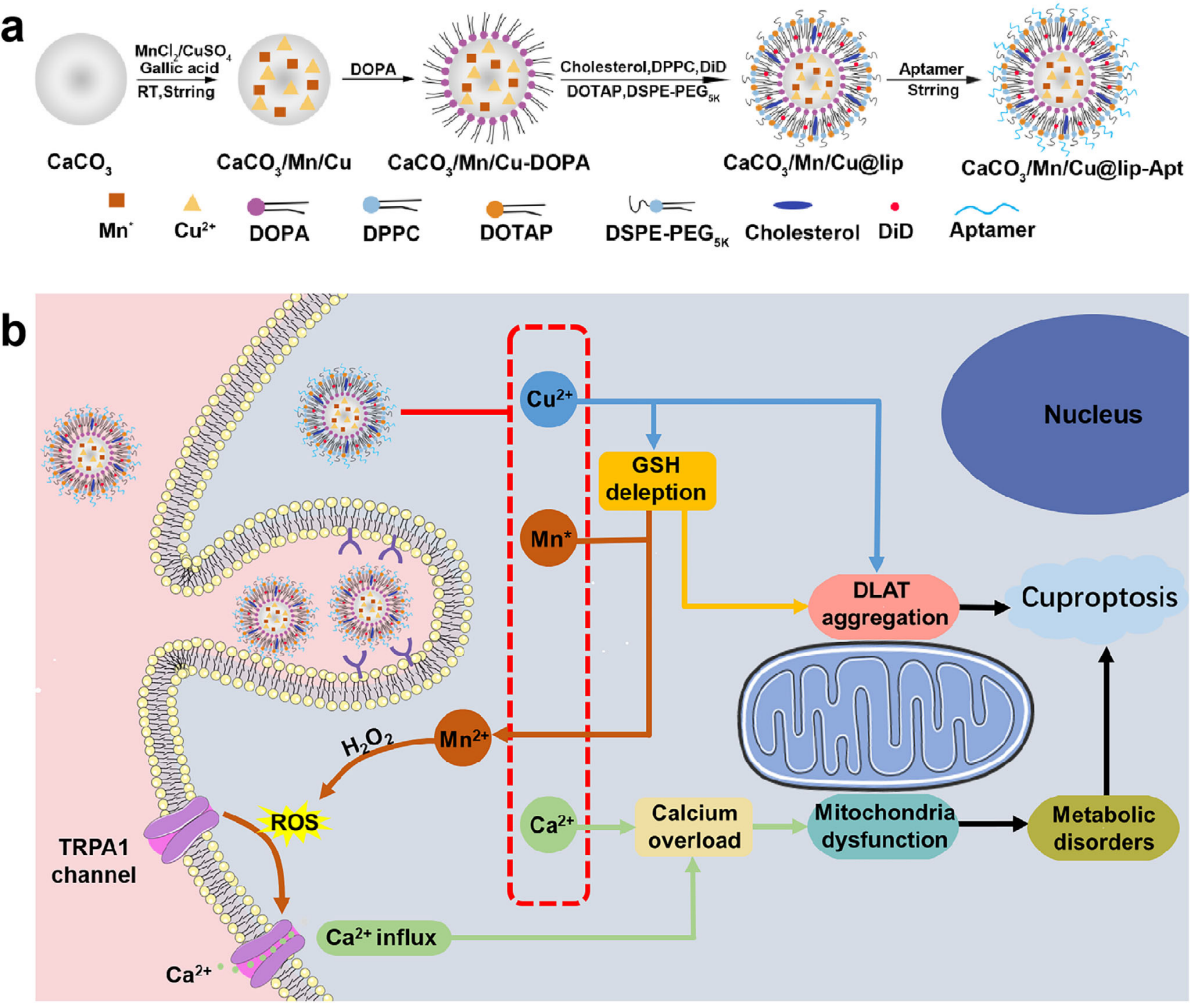
a) Design of CaCO_3_/Mn/Cu@lip‐Apt. b) The mechanism of CaCO_3_/Mn/Cu@lip‐Apt enhancing cuproptosis under the action of calcium and manganese.

In this paper, the synthesis principle of CaCO_3_/Mn/Cu@lip‐Apt is shown in **Figure** **1**. Briefly, CaCO_3_ NPs were first synthesized by the gas diffusion method.^[^ ^24^ ^]^ CaCO_3_ was dispersed into the ethanol solution. Gallic acid (GA) coordinated with Ca^2+^, Cu^2+^, and Mn* to obtain CaCO_3_/Mn/Cu, following formed a lipid layer on the surface through hydrophilic and hydrophobic interactions. Cholesterol was inserted into the lipid component to stabilize the lipid layer, which provided an insertion site for aptamers. In addition, due to its lipid solubility, DiD molecules can be used to label the lipid layer to provide signals for fluorescence imaging analysis.

Under the targeting effect of aptamer on the surface, CaCO_3_/Mn/Cu@lip‐Apt could effectively target MCF‐7 cells, reducing the toxicity to normal cells while increasing the toxicity to tumor cells. The acidic intracellular environment caused CaCO_3_/Mn/Cu@lip‐Apt to decompress, releasing Ca^2+^, Mn*, and Cu^2+^. First, these Ca^2+^ caused calcium overload leading to mitochondrial dysfunction. Second, the high‐valent Mn ions could effectively consume GSH in cells. On the one hand, the resulting Mn^2+^ which could catalyze H_2_O_2_ to generate ROS. This was the Fenton‐like reaction of Mn. ROS could promote the influx of Ca^2+^ through the TRPA1 protein, which further enhanced the calcium overload. It was reported that FDX1 was an upstream regulator of protein fatty acylation for the main mechanism of cuproptosis.^[^ ^3^ ^]^ When the Cu^2+^ interacted with FDX‐1, toxic Cu^+^ was produced and FDX‐1 content was reduced. On the other hand, since GSH was consumed, the amount of chelating Cu^2+^ was reduced, which allowed more Cu^2+^ to interact with FDX‐1. This process generated more Cu^+^, further reducing the FDX‐1 content and increasing the oligomerization of DLAT.^[^ ^3^ ^]^ Finally, the proposed strategy enhanced cuproptosis (Figure 1).

### 2.2 Characterization of CaCO_3_/Mn/Cu@lip‐Apt

**Figure 2:**
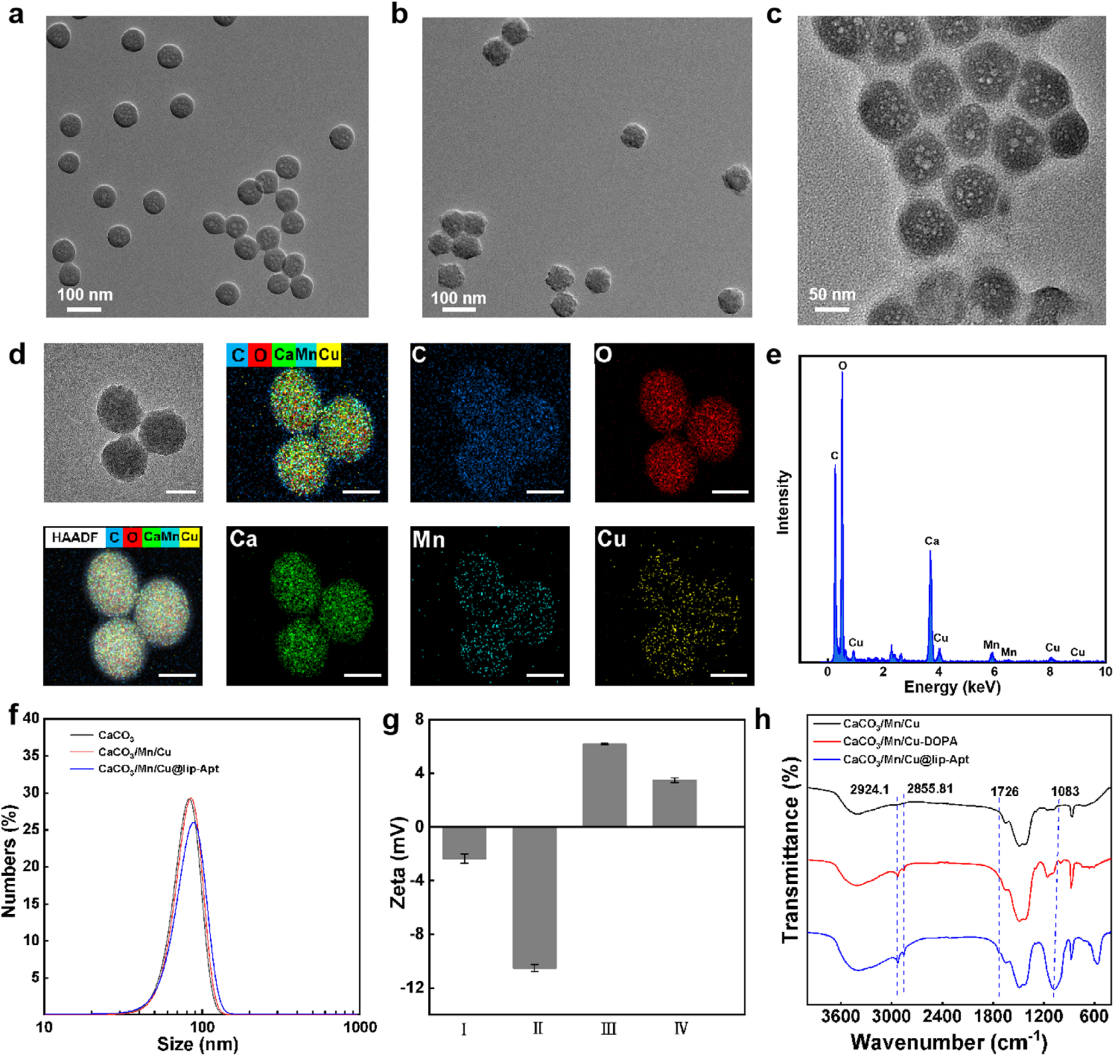
a–c) TEM images of CaCO_3_, CaCO_3_/Mn/Cu, CaCO_3_/Mn/Cu@lip‐Apt. d) Image of CaCO_3_/Mn/Cu, showing the element distribution of C, O, Mn, Ca, and Cu. Scale bar: 50 nm. e) EDS spectrum of CaCO_3_/Mn/Cu. f) DLS pattern. g) Zeta potentials. (I: CaCO_3_, II: CaCO_3_/Mn/Cu, III: CaCO_3_/Mn/Cu@lip, IV: CaCO_3_/Mn/Cu@lip‐Apt) h) FTIR spectra of CaCO_3_/Mn/Cu, CaCO_3_/Mn/Cu‐DOPA and CaCO_3_/Mn/Cu@lip‐Apt.

It could be seen from the transmission electron microscope (TEM) image that the prepared CaCO_3_, CaCO_3_/Mn/Cu, and CaCO_3_/Mn/Cu@lip‐Apt had uniform morphology and good dispersion (**Figure** **2**a‐c). The synthesized CaCO_3_/Mn/Cu@lip‐Apt NPs had the amorphous structure of CaCO_3_, which was confirmed by their X‐ray diffraction pattern (XRD) (Figure S1, Supporting Information).^[^ ^25^ ^]^ The prepared CaCO_3_/Mn/Cu demonstrated distinct elemental signals of C, O, Ca, Mn, and Cu under elemental mapping (Figure 2d), which was also recorded by their energy dispersive spectra (EDS) (Figure 2e). Further using an inductively coupled plasma optical emission spectrometer (ICP‐OES), we found that the Ca, Mn, and Cu, content was 35.76%, 3.104%, and 3.392%, respectively (Figure S2, Supporting Information).

The particle sizes were ≈90 nm, which was consistent with the date of dynamic light scattering (DLS) (Figure 2f). In addition, when these NPs were stored in PBS for 7 days, the particle size of the NPs changed very little, indicating their good stability in PBS. (Figure S3, Supporting Information). According to zeta potential analysis, both CaCO_3_ and CaCO_3_/Mn/Cu were electronegative, which was consistent with literature reports.^[^ ^14^ ^]^ Simultaneously, it was observed that the CaCO_3_/Mn/Cu@lip exhibited a positive charge, which was attributed to the electropositive lipid DOTAP. There was a decrease in the potential of CaCO_3_/Mn/Cu@lip‐Apt compared to CaCO_3_/Mn/Cu@lip, which was caused by the binding of the negatively charged aptamer to the surface (Figure 2g). In addition, the Fourier transform infrared (FT‐IR) spectra peak of the NPs was measured. As shown in Figure 2h, the CaCO_3_/Mn/Cu@lip‐Apt had two absorption peaks at 2924.1 and 2855.81 cm^−1^, corresponding to the saturated and unsaturated glycerides respectively. There was an absorption peak of the ester bond in phospholipids in the range of 1750–1700 cm^−1^, and the absorption peak of the phosphate bond was at 1083 cm^−1^.^[^ ^26^ ^]^ CaCO_3_/Mn/Cu did not show the above absorption peaks. According to the above data, it can be concluded that the phospholipid composed of a lipid layer was successfully modified on the CaCO_3_/Mn/Cu surface. Next, according to the high‐resolution Mn 2p_3/2_ X‐ray photoelectron spectroscopy (XPS) spectra (Figure S4, Supporting Information), the valence states of Mn in the CaCO_3_/Mn/Cu were mainly Mn^4+^ (≈43.48%) and Mn^3+^ (≈19.96%).^[^ ^27^ ^]^ The presence of Mn^4+^ and Mn^3+^ provided the basis for intracellular GSH depletion.^[^ ^28^ ^]^

### 2.3 pH‐Responsivity and Extracellular Release Properties

**Figure 3:**
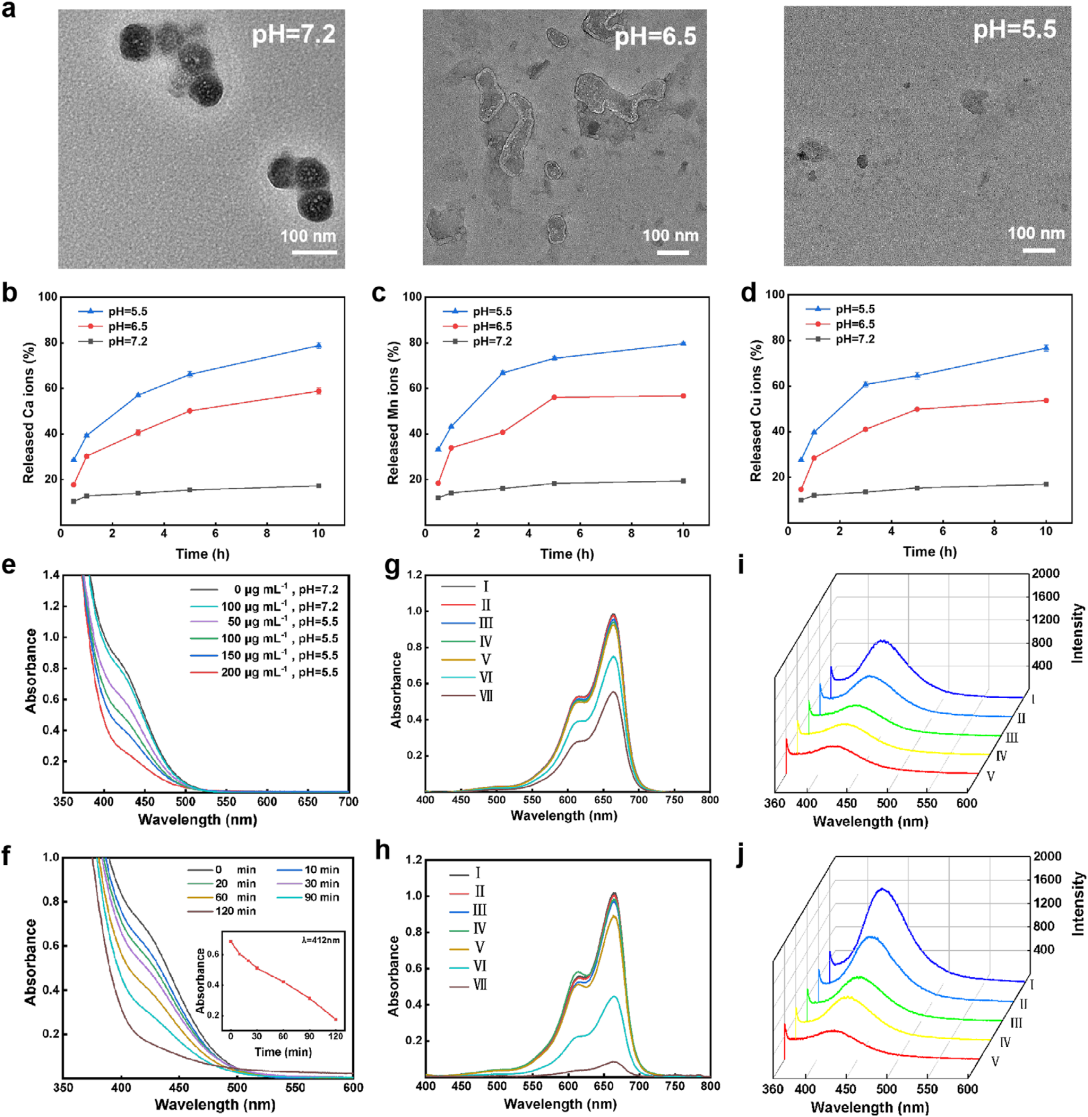
a) TEM image of CaCO_3_/Mn/Cu@lip‐Apt soaked in PBS buffer at pH 5.5, 6.5, and 7.2 for 1 h. b–d) Release of Ca b), Mn c), and Cu d) from the CaCO_3_/Mn/Cu@lip‐Apt at different pH conditions. e) Absorption curves of CaCO_3_/Mn/Cu@lip‐Apt solution with different concentrations reacting with 1 mm GSH. f) Absorption curves of 150 µg mL^−1^ CaCO_3_/Mn/Cu@lip‐Apt solution after reaction with 1 mm GSH for a different time. g) Absorption curves of MB solution after reaction with different samples (I: MB; II: MB+CaCO_3_/Mn/Cu@lip‐Apt; III: MB+H_2_O_2_; IV: MB+CaCO_3_/Mn/Cu@lip‐Apt+H_2_O_2_; V: MB+CaCO_3_/Mn/Cu@lip‐Apt+H_2_O_2_, pH 7.2; VI: MB+CaCO_3_/Mn/Cu@lip‐Apt+H_2_O_2_, pH 6.5; VII: MB+CaCO_3_/Mn/Cu@lip‐Apt+H_2_O_2_ pH 5.5.) h) The absorption spectrum of the new solution formed after GSH was added to the g) solution. i) Fluorescence spectra of the product of the reaction between •OH (generated by CaCO_3_/Mn/Cu@lip‐Apt at different pH) and TPA. (I: TPA+H_2_O_2_+CaCO_3_/Mn/Cu@lip‐Apt, pH 5.5; II: TPA+H_2_O_2_+CaCO_3_/Mn/Cu@lip‐Apt, pH 6.5; III: TPA+H_2_O_2_+CaCO_3_/Mn/Cu@lip‐Apt, pH 7.2; IV: TPA+H_2_O_2_+ CaCO_3_/Mn/Cu@lip‐Apt; V: TPA+H_2_O_2_.) j) Fluorescence spectra of the new solution formed after GSH was added to the i) solution.

We hope that calcium‐based NPs can be broken down by the acidic tumor microenvironment and release its cargo. So, to determine the structural changes of CaCO_3_/Mn/Cu@lip‐Apt at different pH (7.2, 6.5, 5.5), we observed the morphology by TEM. As shown in **Figure** **3**a, CaCO_3_/Mn/Cu@lip‐Apt was ≈90 nm at pH 7.2. When the pH value of 6.5, the structure of CaCO_3_/Mn/Cu@lip‐Apt was destroyed. When the pH value dropped to 5.5, the CaCO_3_/Mn/Cu@lip‐Apt completely decomposed and the spherical structure basically disappeared. Figure 3b–d showed that CaCO_3_/Mn/Cu@lip‐Apt can gradually release Ca^2+^, Mn*, Cu^2+^ in an acidic environment with the increase of reaction time in different pH solutions. Under the condition of pH 5.5, after 10 h reaction, the release rate of metal ions could reach ≈80%, indicating that CaCO_3_/Mn/Cu@lip‐Apt could effectively respond to a slightly acidic environment and release the loaded Ca^2+^, Mn*, and Cu^2+^. CaCO_3_/Mn/Cu@lip‐Apt was incubated in solution with an initial pH of 7.2, 6.5, and 5.5, respectively. The results showed that with the increase of incubation time, the pH value of the solution with an initial pH 5.5 increased the most (Figure S5a, Supporting Information). The pH value of the solution with an initial pH 6.5 increased second, and the pH value of the solution with an initial pH 7.2 did not change significantly. In addition, CaCO_3_/Mn/Cu@lip‐Apt was incubated in a solution with an initial pH 6.5 for a long time, and the pH of the solution could rise to nearly neutral (Figure S5b, Supporting Information). Therefore, CaCO_3_/Mn/Cu@lip‐Apt could effectively respond to acidic environments and could not be degraded in neutral environments, which laid a good foundation for its application in the acidic tumor microenvironment.

Next, we evaluated the ability of CaCO_3_/Mn/Cu@lip‐Apt to deplete GSH and generate •OH. 2‐nitro‐5‐mercaptobenzoic acid was the product of the reaction between GSH and 5,5‐dithiobis (2‐nitrobenzoic acid) (DTNB). It has an absorbance at 412 nm. With this feature, GSH content can be detected by DTNB. After the reaction between GSH and CaCO_3_/Mn/Cu@lip‐Apt (0 µg mL^−1^ or 100 µg mL^−1^, pH 7.2), the absorbance at 412 nm did not change significantly. In the solution with pH 5.5, the absorbance of the solution decreases gradually at 412 nm as the concentration of CaCO_3_/Mn/Cu@lip‐Apt increases (Figure 3e). With the extension of incubation time between CaCO_3_/Mn/Cu@lip‐Apt and GSH, the absorbance of the solution at 412 nm gradually decreased (Figure 3f). After the reaction of CaCO_3_, CaCO_3_/Cu, CaCO_3_/Mn, CaCO_3_/Mn/Cu with the same concentration as GSH, the absorption curve of the solution was compared with that of the pure GSH group (Figure S6a, Supporting Information). It was found that the CaCO_3_ group cannot consume GSH. The GSH consumption of the CaCO_3_/Mn group was higher than that of the CaCO_3_/Cu group. CaCO_3_/Mn/Cu had the highest GSH consumption. This was due to the fact that Cu^2+^ and Mn* formed by the decomposition of NPs can consume GSH.

Next, methylene blue (MB) was used to evaluate the ability of CaCO_3_/Mn/Cu@lip‐Apt to catalyze H_2_O_2_ to generate •OH. As illustrated in Figure 3g, the absorbance at ∼664 nm decreased with the decrease of pH. This may be due to the fact that at low pH, the decomposed NPs produced Mn^2+^ to catalyze H_2_O_2_ to generate •OH (Fenton‐like reaction of manganese), which can oxidize MB, resulting in a decrease in absorbance at ∼664 nm. After adding GSH to the solution, the absorbance showed a significant decrease in the acid solution (Figure 3h). It could be discerned from Figure S6b (Supporting Information) that the absorption curves of MB subsequent to treatment with various NPs declined successively. This was because in the presence of GSH, Cu^+^ generated by the reaction of Cu^2+^ with GSH had stronger Fenton‐like catalytic activity.^[^ ^29^ ^]^

Terephthalic acid (TPA) was known to react with ·OH to form 2‐hydroxyterephthalic acid, a fluorescent product. The quantity of • OH generated was indicated by the fluorescence intensity of 2‐hydroxyterephthalic acid at ≈420 nm. As the pH decreased, the fluorescence intensity of 2‐hydroxyterephthalic acid continued to increase (Figure 3i). It further illustrated that CaCO_3_/Mn/Cu@lip‐Apt decomposed under slightly acidic conditions and catalyzes H_2_O_2_ to generate •OH. After the addition of GSH (Figure 3j), the fluorescence intensity at 420 nm was stronger than that in Figure 3i. The fluorescence intensity of TPA + H_2_O_2_+CaCO_3_/Mn/Cu+GSH was the highest (Figure S6c, Supporting Information). These showed that GSH converted the high‐valent Mn* into Mn^2+^, which promoted the production of •OH. In the presence of GSH, CaCO_3_/Mn/Cu generation catalyzed the formation of the highest amount of ·OH.

### 2.4 In Vitro Cellular Uptake

Subsequently, the ability of CaCO_3_/Mn/Cu@lip‐Apt to enter cells and its entry pathway was investigated by fluorescence microscopy. As shown in Figure S7a (Supporting Information), the red fluorescence in MCF‐7 cells enhanced with increasing incubation time. In contrast, after the cocultivation of MRC‐5 cells and CaCO_3_/Mn/Cu@lip‐Apt, the red fluorescence was not obvious (Figure S7b, Supporting Information). The expression of MUC1 protein in MCF‐7 cells was elevated compared to MRC‐5 cells, leading to a greater accumulation of NPs in MCF‐7 cells by the MUC1‐targeting nucleic acid aptamer, which consequently resulted in stronger fluorescence.^[^ ^30^ ^]^ The above results indicated that CaCO_3_/Mn/Cu@lip‐Apt had a good ability to target MCF‐7 cells.

The entry of CaCO_3_/Mn/Cu@lip‐Apt into cells was studied using three endocytic pathway inhibitors: CPZ, AMI, and M‐β‐CD. Fluorescence microscopy and flow cytometry assay showed that the CPZ and AMI groups were similar to the control group without endocytosis inhibitor treatment, while the fluorescence of M‐β‐CD‐treated cells was significantly weakened (Figure S8, Supporting Information). According to the literature,^[^ ^31^ ^]^ we could conclude that the entry pathway of CaCO_3_/Mn/Cu@lip‐Apt was mainly internalized through clathrin‐dependent endocytosis.

### 2.5 Cytotoxicity and Mechanism

**Figure 4:**
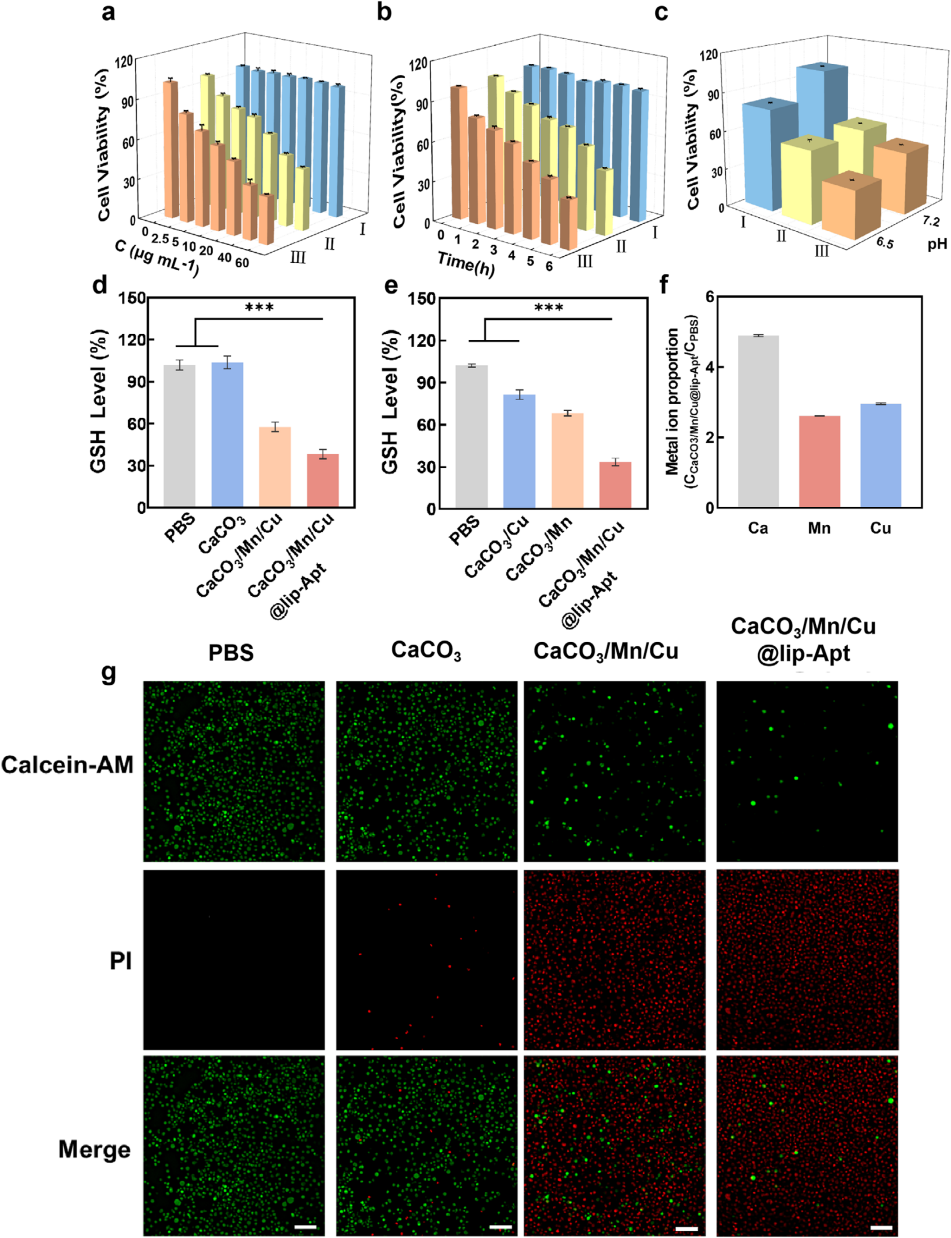
a) Cell viability of MCF‐7 cells treated with different concentrations of CaCO_3_, CaCO_3_/Mn/Cu, and CaCO_3_/Mn/Cu@lip‐Apt. b) When the concentration of CaCO_3_, CaCO_3_/Mn/Cu, and CaCO_3_/Mn/Cu@lip‐Apt was 60 µg mL^−1^, cell viability after culture for different times. c) Cell viability was assessed after incubating cells with CaCO_3_, CaCO_3_/Mn/Cu, and CaCO_3_/Mn/Cu@lip‐Apt at pH levels of 6.5 and 7.2 respectively. a–c), I: CaCO_3_; II: CaCO_3_/Mn/Cu; III: CaCO_3_/Mn/Cu@lip‐Apt. *n* = 3) d) GSH levels in cells were measured after incubation with CaCO_3_, CaCO_3_/Mn/Cu, and CaCO_3_/Mn/Cu@lip‐Apt. e) GSH levels in cells were measured after incubation with CaCO_3_/Cu, CaCO_3_/Mn, and CaCO_3_/Mn/Cu@lip‐Apt. (Data are represented as mean ± SD. p values were calculated via a one‐way ANOVA test in (d and e) ^***^ *p* < 0.001. *n* = 3) f) Changes in intracellular Ca, mMn, and Cu content after CaCO_3_/Mn/Cu@lip‐Apt incubation with MCF‐7 cells for 12 h. (*n* = 3) g) Calcein‐AM (green, living cells) and PI (red, dead cells) fluorescence images of MCF‐7 cells following incubation with different NPs without DiD. Scale bar: 100 µm.

The cytotoxicity of CaCO_3_, CaCO_3_/Mn/Cu, CaCO_3_/Mn/Cu@lip‐Apt was investigated, respectively. MTT results showed that the cell viability after CaCO_3_ treatment remained basically unchanged, while the cell viability of CaCO_3_/Mn/Cu and CaCO_3_/Mn/Cu@lip‐Apt group gradually decreased with the increase of NPs concentration. It could be seen from the results that CaCO_3_/Mn/Cu and CaCO_3_/Mn/Cu@lip‐Apt could kill tumor cells (**Figure** **4**a). Similarly, the cytotoxicity of CaCO_3_/Mn/Cu and CaCO_3_/Mn/Cu@lip‐Apt was time‐dependent (Figure 4b). In addition, Figure S9 (Supporting Information) further proved that CaCO_3_/Mn/Cu@lip‐Apt had higher cytotoxicity than CaCO_3_/Mn and CaCO_3_/Cu.

Subsequently, we evaluated the cell viability of CaCO_3_, CaCO_3_/Mn/Cu, and CaCO_3_/Mn/Cu@lip‐Apt cultured at different pH values (6.5 and 7.2) (Figure 4c). When pH 6.5, compared with CaCO_3_ group, cell viability decreased significantly after being treated with CaCO_3_/Mn/Cu and CaCO_3_/Mn/Cu@lip‐Apt, indicating that CaCO_3_/Mn/Cu and CaCO_3_/Mn/Cu@lip‐Apt can decompose and release Ca^2+^, Mn* and Cu^2+^ under weakly acidic environment. This can promote the production of intracellular ROS, effectively promote the Ca^2+^mediated calcium overload effect, and achieve effective killing of tumors. When pH 7.2, the cell viability of all groups after treatment was higher than that of pH 6.5, indicating that NPs in all groups were relatively stable under neutral conditions.

Next, we evaluated the depletion ability of CaCO_3_/Mn/Cu@lip‐Apt on intracellular GSH content. In Figure 4d, compared with the PBS group, CaCO_3_ had no significant effect on intracellular GSH content. CaCO_3_/Mn/Cu and CaCO_3_/Mn/Cu@lip‐Apt effectively consumed intracellular GSH. In Figure 4e, intracellular GSH levels decreased successively after treatment with CaCO_3_/Cu, CaCO_3_/Mn, and CaCO_3_/Mn/Cu@lip‐Apt. When NPs are internalized by cells, the released high‐valence Mn* reacts with GSH to generate Mn^2+^. After NPs entered the cells, the high‐valent Mn* was released and reacted with GSH, consuming GSH.

As shown in Figure 4f, after incubation of CaCO_3_/Mn/Cu@lip‐Apt and MCF‐7 cells for 12 h, the intracellular Ca content was ≈5 times that of PBS group, the Mn content was ≈2.5 times that of PBS group, and the Cu content was ≈2.8 times that of PBS group. These indicated that CaCO_3_/Mn/Cu@lip‐Apt increased the level of intracellular metal ions.

In addition, Calcein‐AM and PI double fluorescence staining was performed on MCF‐7 cells after different treatments (Figure 4g). Both the PBS and CaCO_3_ groups showed bright green fluorescence, indicating that CaCO_3_ was not highly toxic to cells. In contrast, the CaCO_3_/Mn/Cu group had stronger red fluorescence, and the CaCO_3_/Mn/Cu@lip‐Apt group had the strongest red fluorescence. The calcium overload effect and copper reduction effect enhanced the killing effect of CaCO_3_/Mn/Cu on MCF‐7 cells. CaCO_3_/Mn/Cu@lip‐Apt was more easily internalized by cells than CaCO_3_/Mn/Cu, so it had the strongest inhibitory effect on cells.

DCFH‐DA probe was employed as a detector to study the intracellular ·OH. After CaCO_3_, CaCO_3_/Mn/Cu, and CaCO_3_/Mn/Cu@lip‐Apt were incubated with cells respectively, their ability to generate ROS was evaluated through the DCFH‐DA probe. Through fluorescence imaging in **Figure** **5**a and Figure S10a (Supporting Information), it can be found that the intracellular fluorescence intensity of cells treated with CaCO_3_/Mn/Cu was significantly enhanced. This was because CaCO_3_/Mn/Cu was loaded with Cu^2+^/Mn*, which destroyed the homeostasis of intracellular Ca^2+^, causing cellular oxidative stress and generating ROS. At this time, ROS oxidizes DCFH‐DA to produce DCF with the green fluorescent signal. The fluorescence intensity of cells treated with CaCO_3_/Mn/Cu@lip‐Apt was further enhanced. The corresponding flow cytometry further demonstrated that the fluorescence intensity of DCF increased (Figure 5a), which was consistent with the conclusion of the cell fluorescence imaging data. In addition, flow cytometry was used to analyze the fluorescence intensity of DCF after incubation of CaCO_3_/Mn/Cu@lip‐Apt and MCF‐7 cells at different times. The fluorescence intensity of DCF was positively correlated with that of DiD (versus CaCO_3_/Mn/Cu@lip‐Apt) in cells, and it was time‐dependent. (Figure S11, Supporting Information). In Figures S12 and S13 (Supporting Information), compared with CaCO_3_/Cu or CaCO_3_/Mn, the fluorescence images further demonstrated that CaCO_3_/Mn/Cu could significantly increase the level of intracellular ROS.

Figure 5a) Fluorescence images, corresponding surface plot images and flow cytometry of MCF‐7 cells after incubation with CaCO_3_, CaCO_3_/Mn/Cu, and CaCO_3_/Mn/Cu@lip‐Apt, using DCFH‐DA to evaluate ROS production. Scale bar: 50 µm. b) Fluorescence images, corresponding surface plot images and flow cytometry of MCF‐7 cells incubated with CaCO_3_, CaCO_3_/Mn/Cu, and CaCO_3_/Mn/Cu@lip‐Apt. Scale bar:100 µm. c) Fluorescence image of JC‐1 and corresponding surface plot images of MCF‐7 cells after incubation with CaCO_3_, CaCO_3_/Mn/Cu, and CaCO_3_/Mn/Cu@lip‐Apt (without DiD), respectively. Scale bar: 20 µm. d) Fluorescent images of MCF‐7 cells treated with PBS, CaCO_3_/Mn/Cu@lip‐Apt, and Mito‐Tracker Green under different times, scale bar: 20 µm. In the flow cytometry, PBS group was MCF‐7 cells without any other treatment. 0.5 h group, 1 h group, and 3 h group showed the MCF‐7 cells incubated with CaCO_3_/Mn/Cu@lip‐Apt for 0.5, 1, and 3 h, respectively. e) Intracellular ATP level. (*n* = 3) f) Western blot analysis was performed on the protein expression of cuproptosis‐related proteins (FDX‐1 and DLAT) in MCF‐7 cells. The loading control was β‐actin. g) DLAT immunofluorescence images of cells in different groups. The CaCO_3_/Mn/Cu@lip‐Apt used here did not contain DiD. Scale bar: 20 µm.

**Figure:**
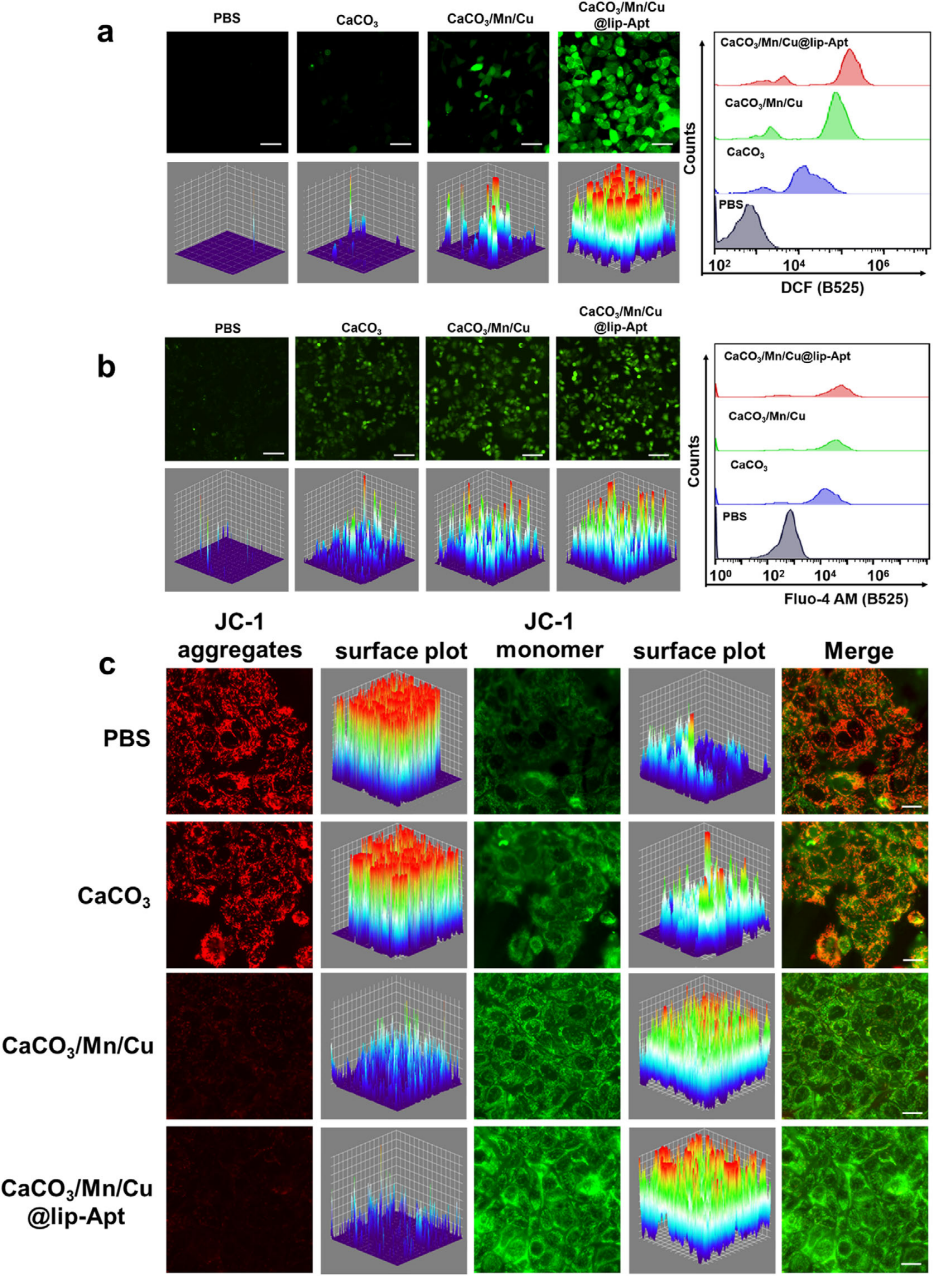

**Figure:**
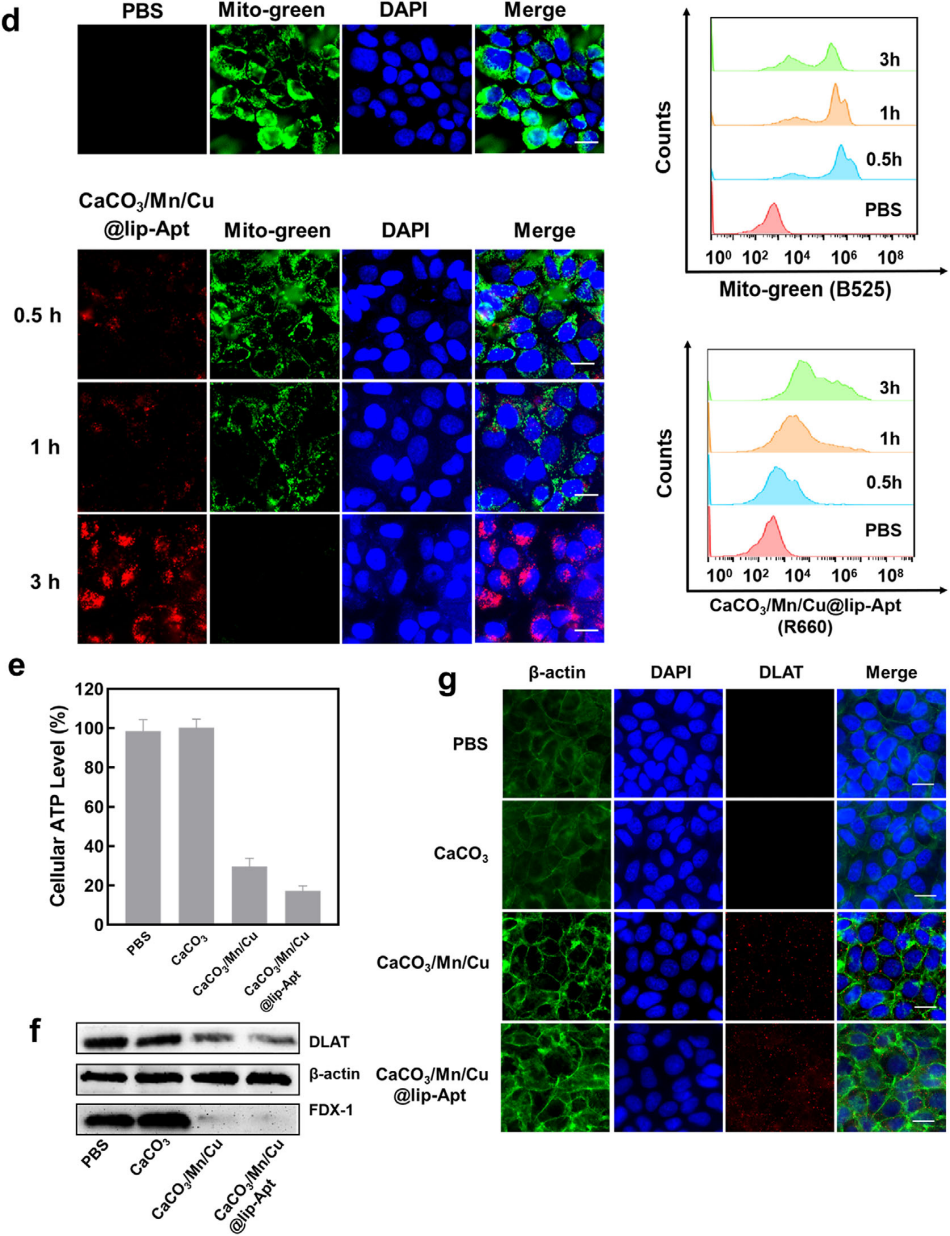

The changes in intracellular Ca^2+^ content were detected by the Fluo‐4AM probe. In Figures S14 and S15 (Supporting Information), compared with CaCO_3_/Cu or CaCO_3_/Mn, the fluorescence images further demonstrated that CaCO_3_/Mn/Cu could significantly increase the level of intracellular Ca^2+^. Compared with the green fluorescence intensity in MCF‐7 cells after CaCO_3_ treatment, the fluorescence intensity of cells treated with CaCO_3_/Mn/Cu and CaCO_3_/Mn/Cu@lip‐Apt was enhanced successively (Figure 5b; Figure S10b, Supporting Information). The corresponding flow cytometry also shows a similar phenomenon. These were due to the catalytic generation of ·OH by Cu^2+^ and Mn* in NPs, which broke the intracellular Ca^2+^ balance. With the extension of incubation time, the fluorescence intensity of DiD (vs CaCO_3_/Mn/Cu@lip‐Apt) and Fluo‐4 AM increased in the cells (Figure S16, Supporting Information).

After verifying intracellular calcium overload, we subsequently examined mitochondrial status. Red fluorescence could be produced by JC‐1 aggregates, which were formed when JC‐1 aggregates in the mitochondrial matrix at high mitochondrial membrane potential. JC‐1 cannot aggregate in the mitochondrial matrix at low mitochondrial membrane potentials, and its monomer (JC‐1 monomer) exhibited green fluorescence. It could be seen that CaCO_3_ basically did not affect the mitochondrial membrane potential (Figure 5c). Compared with CaCO_3_, cells treated with CaCO_3_/Mn/Cu and CaCO_3_/Mn/Cu@‐lip‐Apt both showed stronger green fluorescence and weaker red fluorescence. Moreover, the green fluorescence after CaCO_3_/Mn/Cu@lip‐Apt treatment was the strongest, and the red fluorescence almost disappeared (Figure 5c). So, calcium overload caused by CaCO_3_/Mn/Cu and CaCO_3_/Mn/Cu@lip‐Apt leads to mitochondrial damage. The aptamer on the surface of CaCO_3_/Mn/Cu@lip‐Apt improved the efficiency of internalization by cells, further enhancing mitochondrial damage. These echoed the conclusion of Figure 5b. Flow cytometry showed that with the increase of incubation time between CaCO_3_/Mn/Cu@lip‐Apt and MCF‐7 cells, the proportion of JC‐1 monomer gradually increased, indicating that the mitochondrial membrane potential continued to decrease (Figure S17, Supporting Information). In addition, in Figure S18 (Supporting Information), compared with CaCO_3_/Cu or CaCO_3_/Mn, the fluorescence images further illustrated that CaCO_3_/Mn/Cu@lip‐Apt could significantly reduce mitochondrial membrane potential. Subsequently, we incubated MCF‐7 cells with CaCO_3_/Mn/Cu@‐lip‐Apt for different times and evaluated the impact of NPs on mitochondria using the specific dye Mito‐tracker‐green. The fluorescence image showed that as the incubation time increased, the red fluorescence of CaCO_3_/Mn/Cu@lip‐Apt gradually increased, while the green fluorescence of Mito‐tracker gradually weakened. The green fluorescence almost disappeared after 3 h of incubation (Figure 5d). Moreover, the flow cytometry analysis results were also consistent with the fluorescence pictures. The mitochondrial morphological changes of MCF‐7 cells treated with CaCO_3_/Mn/Cu@lip‐Apt were further observed by Bio‐TEM. The results showed that mitochondrial membrane density increased and mitochondrial ridge decreased or even disappeared (Figure S19, Supporting Information). Ultimately, the measurement of intracellular ATP levels showed that CaCO_3_/Mn/Cu@lip‐Apt notably decreased the ATP content in cells, establishing the necessary conditions for the onset of cuproptosis. (Figure 5e)

It was found that cells treated with CaCO_3_/Mn/Cu and CaCO_3_/Mn/Cu@lip‐Apt reduced the content of FDX‐1 (Figure 5f). In addition, Cu^+^ bound directly to DLAT, causing DLAT oligomerization and cell cuproptosis. Oligomerization of DLAT was characterized by immunofluorescence. As shown in Figure 5g and Figure S20a (Supporting Information), no obvious presence of DLAT was observed in cells treated with PBS or CaCO_3_, while cells treated with CaCO_3_/Mn/Cu and CaCO_3_/Mn/Cu@lip‐Apt showed obvious DLAT focus. Next, in order to investigate whether CaCO_3_/Mn/Cu@lip‐Apt can cause cuproptosis in other kinds of cells, HepG2 cells and MRC‐5 cells were selected to evaluate the effect of cuproptosis. At the same incubation time, HepG2 cells and MRC‐5 treated with CaCO_3_/Mn/Cu@lip‐Apt showed weak red fluorescence of DLAT oligomers (Figures S21 and S22, Supporting Information). The surface of HepG2 cells and MRC‐5 lacked the target protein of the aptamer, resulting in poor toxicity.^[^ ^30^, ^32^ ^]^ The corresponding western blotting data also confirmed the enhancement of cuproptosis (Figure S20b, Supporting Information). All these results indicate that the Cu^2+^ released by NPs could destroy the stability of FDX‐1,^[^ ^3^ ^]^ and the generated Cu^+^ could cause abnormal oligomerization of DLAT, resulting in cuproptosis.

We continued to study mature mice with bone marrow‐derived dendritic cells (BMDC). MCF‐7 cells treated with different NPs were further incubated with BMDC and the maturation of the dendritic cell (DC) was analyzed by flow cytometry (Figure S23, Supporting Information). The maturity rates of CaCO_3_/Mn (13.4%)group and CaCO_3_/Cu (16.2%) group were significantly lower than those of CaCO_3_/Mn/Cu group respectively. In the group of MCF‐7 cells treated with CaCO_3_/Mn/Cu@lip‐Apt, the maturity rate of DC cells was the highest (38.8%). Taken together, these findings suggest that CaCO_3_/Mn/Cu@lip‐Apt‐induced cuproptosis can effectively trigger an immune response.

### 2.6 Animal Experiments

**Figure 6:**
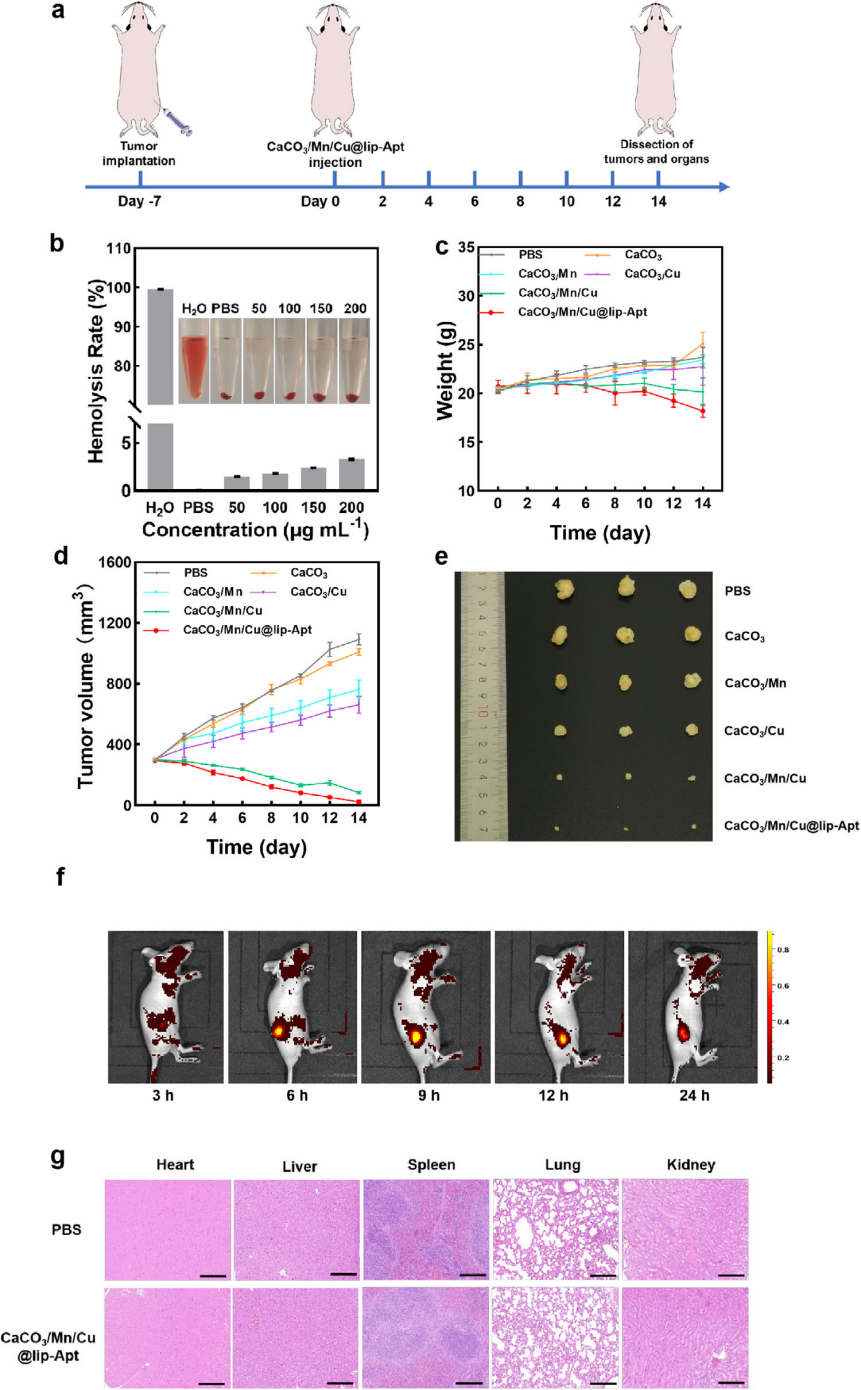
Evaluation of the antitumor activity of CaCO_3_/Mn/Cu@lip‐Apt in vivo. a) Schematic representation of tumor formation and treatment in MCF‐7 tumor‐bearing mice. b) Hemolysis rate of red blood cells treated with different concentrations of CaCO_3_/Mn/Cu@lip‐Apt for 5 h (*n* = 3). As positive and negative controls, there was H_2_O and PBS, respectively (*n* = 3). c) The quantitative relative body weight (*n* = 3). d) The quantitative relative and tumor volume (*n* = 3). e) Photo of tumors collected at the 14th day. f) In vivo fluorescence imaging of MCF‐7 tumor‐bearing mice intravenously injected with CaCO_3_/Mn/Cu@lip‐Apt. g) Histological analysis of the organ using a hematoxylin and eosin stain after various treatments. Scale bar: 200 µm.

The prerequisites for the use of drugs in vivo were good biocompatibility. We used a subcutaneous transplant tumor mice model to evaluate the tumor inhibition effect of CaCO_3_/Mn/Cu@lip‐Apt NPs. The modeling‐treatment process is shown in **Figure** **6**a. First, we selected 5‐week‐old mice and injected PBS or various NPs through the tail vein for three times. The mice were sacrificed after 14 days. Their blood was collected for hemolysis analysis, and organs were used for H&E staining analysis. As shown in Figure 6b, no obvious hemolysis (less than 5%) was observed after CaCO_3_/Mn/Cu@lip‐Apt NPs coexisted with red blood cells at different concentrations (50, 100, 150, 200 µg mL^−1^) for 5 h. To explore the systemic risk of Cu ion accumulation and calcium overload, we studied the biological distribution of Ca, Mn, and Cu in tumor‐bearing mice. Distribution of metallic elements in the viscera of mice after 3 and 6 h intravenous injection. The results showed that the content changes of the three metal elements in the tumor were within a reasonable range and CaCO_3_/Mn/Cu@lip‐Apt has good retention ability in tumor areas (Figure S24a–c, Supporting Information). At the same time, we calculated that the half‐lives (T_1/2_) of Ca, Mn, and Cu in blood circulation were ≈4.617, 4.085, and 1.826 h, respectively (Figure S24d–f, Supporting Information). Moderate cycle time is conducive to the accumulation of CaCO_3_/Mn/Cu@lip‐Apt in tumors. After injections of CaCO_3_/Mn/Cu@lip‐Apt into mice, the weight of the mice fluctuated ≈22 g, which was not significantly different from the weight of mice injected with PBS. This demonstrated that there was very little systemic toxicity to NPs at this level (Figure 6c). All these experimental results showed that CaCO_3_/Mn/Cu@lip‐Apt NPs had good biocompatibility and safety. After the injection of CaCO_3_/Mn/Cu@lip‐Apt NPs, the tumor volume gradually decreased with the increase of time. (Figure 6d,e).

Next, we studied the biodistribution of CaCO_3_/Mn/Cu@lip‐Apt in mice. The distribution of CaCO_3_/Mn/Cu@lip‐Apt in the mice was detected through a small animal imaging system (Figure 6f). The results showed that with the extension of injection time, the tumor site of mice gradually appeared obvious drug aggregation phenomenon. After 9 h, the fluorescent signal on the tumor was the strongest. After 12 h, the fluorescence on the tumor was gradually weakened. CaCO_3_/Mn/Cu@lip‐Apt had good tumor‐targeting effect which was conducive to reducing toxicity to healthy tissues.

Immunogenic cell death (ICD) is a type of cell death that involves the activation of the immune system to fight cancer in an immunocompetent host. ICD is characterized by a series of damage‐associated molecular patterns (DAMPs), including surface‐exposed calretin, secreted ATP, and high mobility histone 1 released by dead cells. These released DAMPs are easily recognized and taken up by DCs, induce DCs maturation, promote T cell activation, and ultimately trigger a powerful anti‐tumor immune response to clear tumor cells.^[^ ^33^ ^]^ Because of the different cell death patterns, we hypothesized that the toxic aggregation of proteins caused by cuproptosis increases exposure to antigenic substances, which in turn activate the immune response via ICD. (Figure S25, Supporting Information)

So, we investigated whether CaCO_3_/Mn/Cu@lip‐Apt can activate the immune response in Balb/c tumor‐bearing mice. Mice tumors were collected and lymphocytes were obtained. The changes of immune cell population in tumor tissues were analyzed by flow cytometry. The results showed that the maturity of DC (CD80^+^, CD86^+^) in tumor tissues of mice treated with CaCO_3_/Mn/Cu@lip‐Apt was ≈4 times as many as that of mice treated with PBS. (Figure S26, Supporting Information) The highly efficient antigen‐presenting ability of DCs will stimulate the proliferation of native T cells and infiltrate into tumor tissues, thus effectively killing tumors. Subsequently, T cell infiltration was analyzed. The results showed that CaCO_3_/Mn/Cu@lip‐Apt significantly increased the infiltration of CD4^+^ T cells and CD8^+^ T cells, which was used in cancer immunotherapy. (Figure S27, Supporting Information)

It is well known that the tumor‐killing ability of CD8^+^ T cells is largely dependent on the production of IFN‐γ.^[^ ^34^ ^]^ Therefore, IFN‐γ secretion was measured, and IFN‐γ secretion was significantly elevated in the CaCO_3_/Mn/Cu@lip‐Apt group (Figure S28a,d, Supporting Information). In addition, CaCO_3_/Mn/Cu@lip‐Apt NPs treatment significantly enhanced the secretion of interleukin‐6 and tumor necrosis factor α (Figure S28b,c,e,f, Supporting Information), further confirming its good therapeutic effect.^[^ ^35^ ^]^

Finally, H&E stains were observed on the heart, liver, spleen, lung, and kidney of mice receiving saline and CaCO_3_/Mn/Cu@lip‐Apt under a light microscope. No significant histological changes were observed in the major organs in each group after treatment (Figure 6g; Figure S29, Supporting Information). The serum biochemical and routine blood indexes of the control group and the material treatment group were all within the normal range (Figure S30, Supporting Information).^[^ ^36^ ^]^ These showed that CaCO_3_/Mn/Cu@lip‐Apt had good biological safety.

## 3 Conclusion

In summary, cuproptosis, as a new way of cell death, provides a new idea for cancer treatment. Research shows that GSH depletion promotes cuproptosis. Because cuproptosis is closely related to mitochondrial metabolism, mitochondrial metabolism disorders can also enhance cuproptosis. Therefore, we designed CaCO_3_/Mn/Cu@lip‐Apt NPs which modified MCF‐7 cell targeting aptamer. CaCO_3_ NPs loaded with Mn* and Cu^2+^. After the NPs are internalized by cells, they decompose and release Cu^2+^, Mn*, and Ca^2+^. Both Mn* and Cu^2+^ effectively consumed GSH in tumor cells, causing GSH depletion and providing conditions for cuproptosis. In addition, the Mn^2+^ which was generated by the reaction of Mn* and GSH can catalyze H_2_O_2_ to generate toxic ROS. The generation of ROS promoted the influx of exogenous Ca^2+^. The inflow of Ca^2+^ and the Ca^2+^ released from NPs enhance the calcium overload effect. Intracellular calcium overload can further cause mitochondrial dysfunction, leading to mitochondrial metabolic disorders and enhanced cuproptosis. In vitro experiments have proven that after CaCO_3_/Mn/Cu@lip‐Apt enters cells, it can significantly increase the intracellular ROS content and Ca^2+^ content, and significantly decrease the GSH content. In immunofluorescence experiments, the formation of DLAT oligomers proved that CaCO_3_/Mn/Cu@lip‐Apt achieved cuproptosis. In vivo experiments proved that CaCO_3_/Mn/Cu@lip‐Apt can effectively inhibit tumor growth. After the tumor tissue is damaged by the NPs, the DAMPs produced by the tumor tissue cause the immune response. Although the results of CaCO_3_/Mn/Cu@lip‐Apt in the treatment of mice are encouraging, there are still great difficulties and challenges in further clinical application. Before this treatment strategy can be extended to clinical use, precise adjustment and dosage optimization for large animal models are needed.

In general, using breast cancer as a model, we studied the mechanism of calcium overload and cuproptosis using CaCO_3_/Mn/Cu@lip‐Apt to accumulate Ca and Cu in the cell. Although this provided a new perspective and method for the study of cuproptosis‐related nanomedicine in clinical treatment, showing great potential for future clinical translation,^[^ ^37^ ^]^ it must be acknowledged that this technology still has a long way to go from the laboratory to the clinical translation.

## 4 Experimental Section

### Synthesis of CaCO_3_@Mn/Cu (CaCO_3_/Mn/Cu)

To synthesize CaCO_3_/Mn/Cu, CaCO_3_ NPs were first prepared by gas diffusion. Triethylamine (TEA,10 µL) and gallic acid (GA, 10 mg mL^−1^, 200 µL) were mixed into CaCO_3_ solution (5 mg mL^−1^ in 4 mL ethanol) containing 35 mg polyvinylpyrrolidone (PVP). The solution was stirred for 5 min at 25 °C. After that, the reaction mixture was added with CuSO_4_ (200 µL, 20 mg mL^−1^) and MnCl_2_ (200 µL, 20 mg mL^−1^), stirring for an additional 45 min. Then, the formed CaCO_3_/Mn/Cu were washed with ethanol by centrifugation at 10 000 rpm.

### Synthesis of CaCO_3_@Mn/Cu@lip‐Aptamer (CaCO_3_/Mn/Cu@lip‐Apt)

For the surface modification of CaCO_3_/Mn/Cu, the CaCO_3_/Mn/Cu (20 mg, 5 mL, ethanol solution) were mixed with 1,2‐dioleoyl‐sn‐glycero‐3‐phosphate (DOPA, 2 mg, 1 mL, chloroform solution) and then sonicated with a water bath for 40 min. Then, the obtained CaCO_3_/Mn/Cu‐DOPA were purified by centrifugation and re‐suspended in chloroform solution (2 mL) containing cholesterol (2 mg), 1,2‐dihexadecanoyl‐sn‐glycero‐3‐phosphocholine (DPPC, 4 mg), fluorescent molecules (DiD, 100 µg), 1,2‐distearoyl‐snglycero‐3phosphoethanolamine‐N‐(methoxy (polyethylene glycol)‐5000) (DSPE‐PEG_5k_, 10 mg) and 1,2‐Dioleoyl‐3‐trimethylammonium‐propane chloride (DOTAP, 12 mg). After being stirred at room temperature overnight, these NPs were removed from chloroform by using a rotary evaporator, and then hydrated with PBS (2 mL) under sonication. These NPs were collected and purified by centrifugation. These NPs and the MCF‐7 aptamer were then stirred overnight in PBS and centrifugally collected, as well as stored at 4 °C for further experiments.

### Statistical Analysis

All data are presented as the mean ± standard deviation. Data analysis was conducted using GraphPad Prism 8. Two‐tailed, unpaired Student's *t*‐tests and one‐way ANOVA were performed to calculate the statistical significance between the different groups, and statistical significance was considered within the groups at a significance threshold of *p* < 0.05.

### Ethics Approval Statement

All animal experiments were approved by the Ethics Committee for Animal Experiments at the Shandong University of Traditional Chinese Medicine (SDUTCM20230922001). The minimum number of animals was used, and every attempt was made to reduce the suffering of the animals.

## Conflict of Interest

The authors declare no conflict of interest.

## Author Contributions

S.L. and W.Y. contributed equally to this work. S.L. and W.Y. performed methodology, validation, formal analysis, and writing. W.Z., J.Z., and Z.L. performed reviewing and editing. Y.G., H.‐Y.C., and J.‐J.X. performed supervision.

## Supporting information



Supporting Information

## Data Availability

The data that support the findings of this study are available in the supplementary material of this article.

## References

[1] a) Z. N. Baker , P. A. Cobine , S. C. Leary , Metallomics 2017, 9, 1501; 28952650 10.1039/c7mt00221a PMC5688007

[2] a) L. Y. Chen , J. X. Min , F. D. Wang , Sig. Transduct Target Ther. 2022, 7, 378; 10.1038/s41392-022-01229-y PMC9681860 36414625

[3] P. Tsvetkov , S. Coy , B. Petrova , M. Dreishpoon , A. Verma , M. Abdusamad , J. Rossen , L. Joesch‐Cohen , R. Humeidi , R. D. Spangler , J. K. Eaton , E. Frenkel , M. Kocak , S. M. Corsello , S. Lutsenko , N. Kanarek , S. Santagata , T. R. Golub , Science 2022, 375, 1254. 35298263 10.1126/science.abf0529 PMC9273333

[4] a) D. L. Tang , X. Chen , G. Kroemer , Cell Res. 2022, 32, 417; 35354936 10.1038/s41422-022-00653-7 PMC9061796

[5] Y. Yang , M. Li , G. Chen , S. Liu , H. Guo , X. Dong , K. Wang , H. Geng , J. Jiang , X. Li , Coord. Chem. Rev. 2023, 495, 215395.

[6] V. Oliveri , Front. Mol. Biosci. 2022, 9, 841814. 35309510 10.3389/fmolb.2022.841814 PMC8931543

[7] T. Xing , L. Li , Y. R. Chen , G. D. Ju , G. L. Li , X. Y. Zhu , Y. B. Ren , J. Zhao , Z. L. Cheng , Y. Li , D. Xu , J. Liang , Cell Rep. Med. 2023, 4, 101264. 37939712 10.1016/j.xcrm.2023.101264 PMC10694624

[8] A. Steinbrueck , A. C. Sedgwick , J. T. Brewster , K. C. Yan , Y. Shang , D. M. Knoll , G. I. Vargas‐Zúñiga , X. P. He , H. Tian , J. L. Sessler , Chem. Soc. Rev. 2020, 49, 3726. 32525153 10.1039/c9cs00373h

[9] a) X. Li , Y. Cui , T. Zhou , J. Li , P. Lu , L. Yuwen , L. Wang , L. Weng , Chem. Eng. J. 2023, 472, 144951;

[10] a) W. Y. Zhang , Z. Y. Li , X. L. Lun , Y. S. Guo , ACS Appl. Mater. Interfaces 2025, 17, 725; 10.1021/acsami.4c18859 39679901

[11] a) W. X. Li , L. Sun , X. F. Zheng , F. Li , W. Y. Zhang , T. Li , Y. S. Guo , D. P. Tang , Anal. Chem. 2023, 95, 9654; 37307415 10.1021/acs.analchem.3c01416

[12] a) Y. X. Wu , D. L. Zhang , X. X. Hu , R. Z. Peng , J. B. Li , X. B. Zhang , W. H. Tan , Angew. Chem., Int. Ed. 2021, 60, 12569; 10.1002/anie.202103027 33739576

[13] a) L. L. Wu , Y. D. Wang , X. Xu , Y. L. Liu , B. Q. Lin , M. X. Zhang , J. L. Zhang , S. Wan , C. Y. Yang , W. H. Tan , Chem. Rev. 2021, 121, 12035; 33667075 10.1021/acs.chemrev.0c01140

[14] a) M. Chang , Z. Hou , D. Jin , J. Zhou , M. Wang , M. Wang , M. Shu , B. Ding , C. Li , J. Lin , Adv. Mater. 2020, 32, 2004647; 10.1002/adma.202004647 32945002

[15] a) H. Kong , Q. Chu , C. Fang , G. Cao , G. Han , X. Li , Adv. Sci. 2021, 8, 2100241; 10.1002/advs.202100241 PMC8292872 34032026

[16] a) X. Y. Ni , W. H. Shi , Y. X. Liu , L. K. Yin , Z. X. Guo , W. Zhou , Q. L. Fan , Small 2022, 18, 2200152; 10.1002/smll.202200152 35398988

[17] a) C. Wang , T. Li , Z. Wang , Y. Li , Y. Liu , M. Xu , Z. Zhang , Y. Deng , L. Cai , C. Zhang , C. Li , J. Nanobiotechnol. 2023, 21, 465; 10.1186/s12951-023-02220-7 PMC10694906 38049882

[18] a) C. Dong , X. Dai , X. Wang , Q. Lu , L. Chen , X. Song , L. Ding , H. Huang , W. Feng , Y. Chen , M. Chang , Adv. Mater. 2022, 34, 2205680; 10.1002/adma.202205680 36106691

[19] a) J. K. Fu , T. Li , Y. C. Zhu , Y. Q. Hao , Adv. Funct. Mater. 2019, 29, 1906195;

[20] R. Zhang , C. G. Wang , Y. K. Guan , X. M. Wei , M. Y. Sha , M. R. Yi , M. Jing , M. Z. Lv , W. Guo , J. Xu , Y. Wan , X. M. Jia , Z. F. Jiang , Cell. Mol. Immunol. 2021, 18, 1222. 33767434 10.1038/s41423-021-00669-w PMC8093200

[21] a) S. S. Wan , Q. Cheng , X. Zeng , X. Z. Zhang , ACS Nano 2019, 13, 6561; 31136707 10.1021/acsnano.9b00300

[22] N. Takahashi , H.‐Y. Chen , I. S. Harris , D. G. Stover , L. M. Selfors , R. T. Bronson , T. Deraedt , K. Cichowski , A. L. Welm , Y. Mori , G. B. Mills , J. S. Brugge , Cancer Cell 2018, 33, 985. 29805077 10.1016/j.ccell.2018.05.001 PMC6100788

[23] a) J. J. Hu , L. Yuan , Y. Zhang , J. Kuang , W. Song , X. Lou , F. Xia , J. Yoon , Angew. Chem., Int. Ed. 2024, 63, e202317578; 10.1002/anie.202317578 38192016

[24] Z. Dong , L. Feng , Y. Hao , M. Chen , M. Gao , Y. Chao , H. Zhao , W. Zhu , J. Liu , C. Liang , Q. Zhang , Z. Liu , J. Am. Chem. Soc. 2018, 140, 2165. 29376345 10.1021/jacs.7b11036

[25] a) C. C. Xue , M. H. Li , Y. Zhao , J. Zhou , Y. Hu , K. Y. Cai , Y. Zhao , S. H. Yu , Z. Luo , Sci. Adv. 2020, 6, eaax1346; 32494659 10.1126/sciadv.aax1346 PMC7190311

[26] X. Zhuang , T. Wu , Y. Zhao , X. Hu , Y. Bao , Y. Guo , Q. Song , G. Li , S. Tan , Z. Zhang , J. Controlled Release. 2016, 228, 26. 10.1016/j.jconrel.2016.02.035 26921522

[27] Y. Zhao , R. Li , J. Sun , Z. Zou , F. Wang , X. Liu , ACS Nano 2022, 16, 5404. 35384646 10.1021/acsnano.1c09008

[28] G. Guan , C. Zhang , H. Liu , Y. Wang , Z. Dong , C. Lu , B. Nan , R. Yue , X. Yin , X. B. Zhang , G. Song , Angew. Chem., Int. Ed. 2022, 134, e202117229. 10.1002/anie.202117229 35460321

[29] a) B. Liu , Y. L. Bian , S. Liang , M. Yuan , S. M. Dong , F. He , S. l. Gai , P. P. Yang , Z. Y. Cheng , J. Lin , ACS Nano 2022, 16, 617; 34957819 10.1021/acsnano.1c07893

[30] a) T. A. Mir , J. H. Yoon , N. G. Gurudatt , M. S. Won , Y. B. Shim , Biosens. Bioelectron. 2015, 74, 594; 26190471 10.1016/j.bios.2015.07.012

[31] Y.‐F. Wang , C. Zhang , K. Yang , Y. Wang , S. Shan , Y. Yan , K. A. Dawson , C. Wang , X.‐J. Liang , Natl. Sci. Rev. 2021, 8, nwab068. 34691676 10.1093/nsr/nwab068 PMC8288177

[32] P. Wu , Y. Gao , H. Zhang , C. Cai , Anal. Chem. 2012, 84, 7692. 22925013 10.1021/ac3015164

[33] D. Ding , X. Jiang , Small Methods 2023, 7, 2300354. 10.1002/smtd.202300354 37191336

[34] M. E. Hoekstra , L. Bornes , F. E. Dijkgraaf , D. Philips , I. N. Pardieck , M. Toebes , D. S. Thommen , J. van Rheenen , T. N. M. Schumacher , Nat. Cancer 2020, 1, 291. 32566933 10.1038/s43018-020-0036-4 PMC7305033

[35] a) X. Hu , Y. Shui , H. Hirano , K. Kusano , W. Z. Guo , M. Fujino , X. K. Li , Cancer Immunol., Immunother. 2023, 72, 719. 36053290 10.1007/s00262-022-03276-4 PMC10992518

[36] J. Zheng , H. Ge , M. Guo , T. Zhang , Q. Hu , Q. Yao , X. Peng , Small 2024, 20, 2304407. 10.1002/smll.202304407 37880907

[37] a) Y. Liao , D. Wang , C. Gu , X. Wang , S. Zhu , Z. Zheng , Z. Gu , Nat. Nanotechnol. 2024, 19, 1892. 39300223 10.1038/s41565-024-01784-1

