# Supplementary material for: Nanoenhanced‐Cuproptosis Results From the Synergy of Calcium Overload and GSH Depletion with the Increasing of Intracellular Ca/Mn/Cu Ions
Source: Adv Sci (Weinh). 2025 Feb 10;12(13):2412067. doi: 10.1002/advs.202412067 (PMC11967785; doi:10.1002/advs.202412067)
Supplement: Supplementary file 1 — Supporting Information [file ADVS-12-2412067-s001.docx]

Supporting Information

Nanoenhanced-cuproptosis Results from the Synergy of Calcium Overload and GSH Depletion with the Increasing of Intracellular Ca/Mn/Cu Ions

Shiwei Liu^#1^, Wennan Yan^#1^, Wenyue Zhang^1^, Ji Zhang^1^, Ziyi Li^1^, Yingshu Guo*^1^, Hong-Yuan Chen^2^, Jing-Juan Xu*^2^

S. Liu, W. Yan, W. Zhang J. Zhang, Z. Li, and Prof. Y. Guo

^1^School of Chemistry and Chemical Engineering, Qilu University of Technology (Shandong Academy of Sciences), Jinan 250353, China.

E-mail: yingshug@126.com

^#^Shiwei Liu and Wennan Yan contributed equally to this work.

Prof. H. Chen and Prof. J. Xu

^2^State Key Laboratory of Analytical Chemistry for Life Science, School of Chemistry and

Chemical Engineering, Nanjing University, Nanjing 210023, China.

E-mail: xujj@nju.edu.cn

Experimental Section

**Materials and reagents**

Ammonia bicarbonate (NH_4_HCO_3_), calcium chloride dehydrate (CaCl_2_·2H_2_O), gallic acid (GA), polyvinylpyrrolidone (PVP K30), PBS (pH 7.4, 10 mM) and the aptamer (5’‑cholesterol-GCAGTTGATCCTTTGGATACCCTGG‑3’) ^[1,2]^ was obtained from Sangon Biotech (Shanghai) Co., Ltd.. Copper sulfate (CuSO_4_), manganese chloride (MnCl_2_), 5,5-dithiobis (2-nitrobenzoic acid) (DTNB), 1,2-dihexadecanoyl-sn-glycero-3-phosphocholine (DPPC), methylene blue (MB), terephthalic acid (TPA), dimethyl sulfoxide (DMSO), 1,2-dioleoyl-sn-glycero-3-phosphate (DOPA), methanol, skim milk, reduced glutathione (GSH), 1,2-distearoyl-snglycero-3phosphoethanolamine-N-(methoxy (polyethylene glycol)-5000) (DSPE-PEG_5k_), cholesterol, 1,2-Dioleoyl-3-trimethylammonium-propane chloride (DOTAP), triethylamine (TEA), chlorpromazine (CPZ), amiloride (AMI), and methyl-β-cyclodextrin (M-β-CD) were purchased from Macklin Inc.. 2,7-dichlorofluorescein diacetate (DCFH-DA), Calcein/PI cell viability/cytotoxicity assay kit, methyl thiazolyl tetrazolium (MTT), DAPI, and DID were purchased from Beyotime Biotechnology. Dulbeccos modified Eagles medium (DMEM), trypsin (0.25% EDTA), antibiotics (penicillin-streptomycin, PS), cell lysis buffer for Western and IP without inhibitors, PMSF, BCA protein assay kit, TBST (20x), stripping buffer, anti-FDX-1 polyclonal antibody, anti-β-actin monoclonal antibody, anti-DLAT polyclonal antibody, GSH test kit were purchased from Beijing Solarbio Science & Technology Co., Ltd.. Ethanol and hydrogen peroxide (H_2_O_2_, 30%) were obtained from Sinopharm Chemical Reagent Co., Ltd.. Color PAGE Gel Rapid Preparation Kit, PVDF (0.22 μm, 0.45 μm) were purchased from Epizyme Biotech. Hypersensitive ECL chemiluminescence kit, tris-glycine SDS-PAGE running buffer, tris-glycine transfer buffer were provided from Wuhan Servicebio Technology Co., Ltd.. Fetal bovine serum (FBS) was provided from Biological Industries. All chemical reagents were used directly without further purification.

**Equipment**

Transmission electron microscopy (TEM) was recorded using a JEOL JEM2100. Ultraviolet–visible absorption spectra were obtained using an Agilent Cary 60 UV-vis Spectrophotometer. Size and zeta potential data were collected with a Malvern Zeta sizer Nano ZS90. X-ray photoelectron spectroscopy (XPS) analysis was recorded by Thermo Scientific™ ESCALAB™ Xi+ to determine the valence states of the Mn/Cu component of CaCO_3_/Mn/Cu. The fluorescence signals were detected and recorded by Hitachi FL-4700. The Fourier transform infrared (FTIR) spectrum was detected by Bruker TENSOR27 FTIR spectrophotometer. The fluorescence images were obtained using Nikon Ti2-E microscope. The amount of fluorescence determined by flow cytometry (Beckman Coulter, CytoFLEX). Mice imaging obtained from IVIS Lumina III imaging system (PerkinElmer).

**Ion Release of** **CaCO_3_/Mn/Cu@lip-Apt**

CaCO_3_/Mn/Cu@lip-Apt was added into PBS solutions (pH=5.5, 6.5 and 7.2, respectively), and the concentration of CaCO_3_/Mn/Cu@lip-Apt was 200 μg mL^-1^. The change in pH was record. The supernatant was taken at different time and the ion content was measured by ICP.

**Evaluation of GSH Depletion Ability of CaCO_3_/Mn/Cu@lip-Apt**

To evaluate the ability of CaCO_3_/Mn/Cu@lip-Apt to consume glutathione, different contents (50, 100, 150, 200 μg·mL^-1^) of CaCO_3_/Mn/Cu@lip-Apt were mixed with 1 mM GSH at 37 °C for 60 min. Then DTNB (100 μL, 2.5 mg·mL^-1^) was added to the solution and mixed for 5 min. The absorption spectrum was recorded and compared with the characteristic absorption peak at 412 nm.

**Extracellular •OH Generation**

The degradation of MB was used to evaluate the formation of ROS. CaCO_3_/Mn/Cu@lip-Apt was added into PBS solutions (pH=5.5, 6.5 and 7.2, respectively). Afterwards, the previous mentioned solution was mixed with 10 μL of MB (100 μg mL^-1^) and 90 μL of H_2_O_2_ (100 mM) before being incubated for 20 min. Then, by using UV-vis spectrophotometry, the absorbance change of MB at 664 nm was measured. Finally, adding GSH (100 μL, 10 mM) to the mixture, the UV absorption was measured 15 min later. Different pH (7.2, 6.5, 5.5) of CaCO_3_/Mn/Cu@lip-Apt solutions (800 μL, 100 μg mL^-1^) and H_2_O_2_ (100 μL, 100 mM) were mixed with TPA (200 μL, 5 mM) for 20 min. The fluorescence intensity of the solution at ~420 nm was then measured under the excitation of 315 nm. Finally, GSH (100 μL, 10 mM) was mixed with the solution and the fluorescence intensity.

**Detection of GSH**

First, the cells had to be collected in order to determine the GSH content. After washing 2-3 times with PBS, the cells were resuspended by adding reagent I from the GSH test kit. Eventually, the supernatant was collected for detection after centrifuging for 10 min at 10,000 rpm. After the reaction, mix the solution (20 μL) with reagent III (40 μL) and reagent II (140 μL) from the GSH test kit. A microplate reader (K3 touch, Thermo) was using determine the absorbance at 412 nm after mixing and holding for 2 min.

**Cell Culture**

MCF-7 cells, MRC-5 and HepG2 cells were provided by Procell Life Technology (Wuhan, China). All cells were cultured in DMEM medium containing 1% penicillin-streptomycin and 10% fetal calf serum. All cells were cultured at 37°C in a CO_2_ incubator with 5% CO_2_.

**Cell Viability Assays**

The cytotoxicity of MCF-7 cells was detected by MTT method. The cytotoxicities of PBS, CaCO_3_, CaCO_3_/Mn/Cu, and CaCO_3_/Mn/Cu@lip-Apt against MCF-7 cells were measured for comparison. Incubate 96-well plates containing MCF-7 cells at 37°C in a CO_2_ incubator with 5% CO_2_ for 12 h. 20 μL CaCO_3_/Mn/Cu@lip-Apt solution was added to each well. The 96-well plate was then cultured in an incubator for 6 h. After adding MTT solution (100 µL, 5 mg mL^-1^) to each well, the cells were incubated for 4 h. After removing the supernatant from the 96-well plate, 100 μL of DMSO was added to each well. The absorbance of the sample at 492 nm was then recorded

**Cellular Uptake Assay**

MCF-7 cells were planted into confocal dishes and incubated overnight to achieve adherence, other cells were also treated as above. Subsequently, 30 μL CaCO_3_/Mn/Cu@lip-Apt solution was added to each dish and incubated for 0.2, 1, and 2 h respectively. After removing the culture medium from the dish, the cells were washed with PBS, and the nuclei were stained with DAPI. Ultimately, the cells were observed by fluorescence microscopy.

**Endocytic Pathways**

The endocytosis routes of NPs were detected using CPZ, AMI, and M-β-CD as endocytosis inhibitors. First, MCF-7 cells were treated for 1 h with the endocytosis inhibitors (CPZ, 30 μg mL^-1^; AMI, 100 μg mL^-1^; M-β-CD, 10 μg mL^-1^). Then, the inhibitor was aspirated, and CaCO_3_/Mn/Cu@lip-Apt was added and incubated for 3 h. Finally, the cells were observed by fluorescence microscopy and the fluorescence intensity changes of the cells were recorded using a flow cytometer.

**Intracellular ROS Detection**

DCFH-DA was used to evaluate the ability of CaCO_3_, CaCO_3_/Mn/Cu and CaCO_3_/Mn/Cu@lip-Apt to produce ROS in cells. First, MCF-7 cells were cultured overnight using confocal dishes. PBS, CaCO_3_, CaCO_3_/Mn/Cu and CaCO_3_/Mn/Cu@lip-Apt were added to the culture dish respectively, and the cells were continued to be cultured for 6 h. After washing the cells 3 times with PBS, adding DCFH-DA (10 μM) to the confocal dish, the cells were incubated for 30 min. At the end of the incubation, the intracellular fluorescence was monitored using fluorescence microscope.

**Observation of Mitochondrial Morphology**

MCF-7 cells were seeded into 6-well plates at a density of 1×10^6^ per well for 12 h. Subsequently, the cells were treated with PBS or CaCO_3_/Mn/Cu@lip-Apt for 24 h. Then, the cells were collected and fixed by electron microscope fixative and observed by Bio-TEM.

**Live/dead Cell Staining Assays**

In order to observe the killing of cells by different samples, the cells after treatment with different samples were stained using Calcein/PI cell viability/cytotoxicity assay kit, observing cells by fluorescence microscope after 30 min. After incubation, live cells and dead cells exhibited

**Evaluation of DLAT Aggregation**

Above all, MCF-7 cells were cultured overnight using confocal dishes. PBS, CaCO_3_, CaCO_3_/Mn/Cu and CaCO_3_/Mn/Cu@lip-Apt were added to the culture dish respectively, and the cells were continued to be cultured for 6 h. Then the cells were incubated with PBS, CaCO_3_, CaCO_3_/Mn/Cu and CaCO_3_/Mn/Cu@lip-Apt for 12 h respectively. Afterwards, 4% paraformaldehyde was used to fix the cells, then TX-100 was used to improve cell permeability. After washing by PBS, the cells incubated with DLAT antibody overnight at 4 ℃ The secondary antibody continued to incubate with cells for 1 h at 25 ℃. Then cells were treated with β-actin-tracker for 0.5 h and then incubated with DAPI for 15 min. Lastly, fluorescence microscopy was used to image. HepG2 cells and MRC-5 cells were treated by the same methods as described above.

**Western Blot Analysis**

The lysed cells were centrifuged by refrigerated centrifuge (4 °C, 12,000 rpm), and the total protein of supernatant was quantified using the BCA kit. To separate protein components, 20 μg of sample was added to each well of 10% SDS-PAGE. After electrophoresis, the protein was transferred to a PVDF membrane (0.45 μm) by electroblotting (Bio-Rad). Anti-DLAT monoclonal antibody incubated with membrane overnight at 4 °C after being blocked for 1 h with 5% skim milk solution. After overnight incubation with the primary antibody, the membranes were washed, HRP-labeled anti-rabbit secondary antibody was added and incubated for 1 h at 25 °C. The bands were detected using a Hypersensitive ECL chemiluminescence kit and imaged by Western blotting and nucleic acid gel imaging analysis system (FluorChem HD2). The detection of β-actin was consistent with the above steps.Meanwhile, in order to detect FDX-1, the PVDF membrane utilized for electroblotting had pore sizes of 0.22 μm. The above detection method was used for imaging detection.

**BMDCs Differentiation**

Beijing Vital River Laboratory Animal Technology Co., Ltd. provided the female Balb/c mice. All mice experimental protocols were approved by the Ethics Committee of Shandong University of Traditional Chinese Medicine (SDUTCM20230922001). Mouse bone marrow cells were obtained from the tibias and femurs of Babl/c mice. The cells were cultured in RPMI-1640 medium containing recombinant mouse granulocyte-macrophage colony stimulating factor and interleukin-4. The suspended and semi-suspended cells were collected on day 7 as differentiated DCs for subsequent experiments.

To investigate BMDCs activation, the transwell insert containing CaCO_3_/Mn/Cu@lip-Apt treated tumor cells was transferred to the culture well of BMDMs for 48 h coincubation.^[3]^ DCs were washed with PBS and stained with FITC-conjugated anti-mouse CD80 and APC-conjugated anti-mouse CD86 for phenotype analysis by flow cytometry. Data were analyzed using FlowJo software.

**Tumor Models**

MCF-7 cells (100 μL, 1×10^6^ cells) were subcutaneously injected into the right side of mice to establish the MCF-7 tumor-bearing mice model. Body weight and tumor size were measured every 2 days. For the experiment, the mice were divided into 4 groups at random. When the tumor volume grew to ~200 mm^3^, different NPs (200 μg mL^-1^, 100 μL) were injected into the tail vein. After 14 days, the mice were killed, and the tumor tissues as well as the primary organs (heart, liver, spleen, lung, and kidney) were taken out for histological.

**Hemolysis Assay**

0.5 mL of mice blood was mixed with 1 mL of PBS and centrifuged at 4000 rpm for 4 min in order to separate red blood cells (RBCs) from the solution. After washing 3-5 times with PBS, dilute the purified red blood cells to 4 mL. Subsequently, 1.2 mL PBS with varying doses of CaCO_3_/Mn/Cu@lip-Apt (50, 100,150 and 200 μg mL^-1^) was mixed with 0.3 mL of the diluted RBC suspension. Samples were incubated at 37 °C for 1 h. After the incubation, the supernatant was extracted by centrifugation and the absorbance at 545 nm was recorded. The positive control group was treated with deionized water, while the negative control group was treated with PBS.

**Histology Analysis**

Mice treated with PBS and NPs were selected, and their hearts, livers, spleens, lungs, and kidneys were cut out and preserved in formalin solution. The fixed tissue was then embedded in paraffin, sectioned, and finally stained with H&E.

**Fluorescence Imaging of the Mice Model**

To study the targeting of the material, the distribution of fluorescence throughout the body of tumor-bearing mice was investigated. the tumor-bearing mice were intratumorally injected with CaCO_3_/Mn/Cu@lip-Apt. After that, fluorescence images were acquired at the predesigned time points.

**In *vivo* Analysis of Immune Cells**

To assess DC maturation ratio in *vivo*, single-cell suspensions were incubated with FITC-conjugated anti-mouse CD80 and APC-conjugated anti-mouse CD86 antibodies at 4 °C for 45 min.^[4]^ Flow cytometry was used to detect and quantify the mature DCs. Changes in the distribution of CD4^+^ and CD8^+^ T cells within tumor tissues were analyzed using, anti-CD4-FITC, and anti-CD8-APC antibodies.

**Enzyme-linked Immunosorbent Assay (ELISA)**

After various treatments, the collected blood samples were naturally solidified at 4 °C for 2 h and centrifuged at 2000 g for 20 min at 4 °C to obtain the blood serum. Blood biochemistry and cytokine levels were determined using ELISA kits, according to the manufacturer’s protocol.


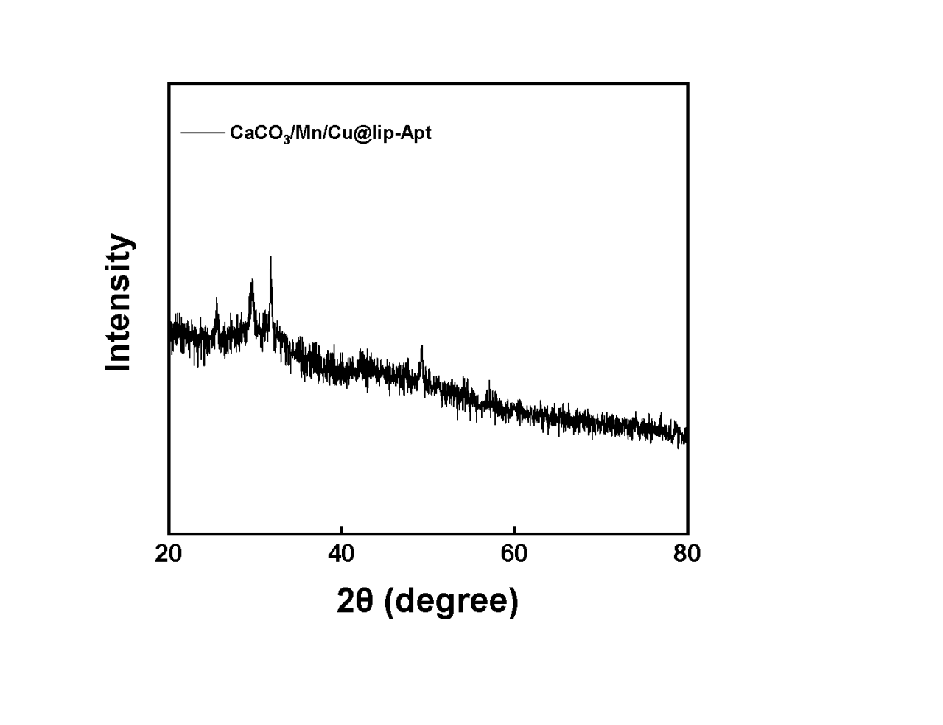


**Figure S1.** XRD patterns of CaCO_3_/Mn/Cu@lip-Apt.


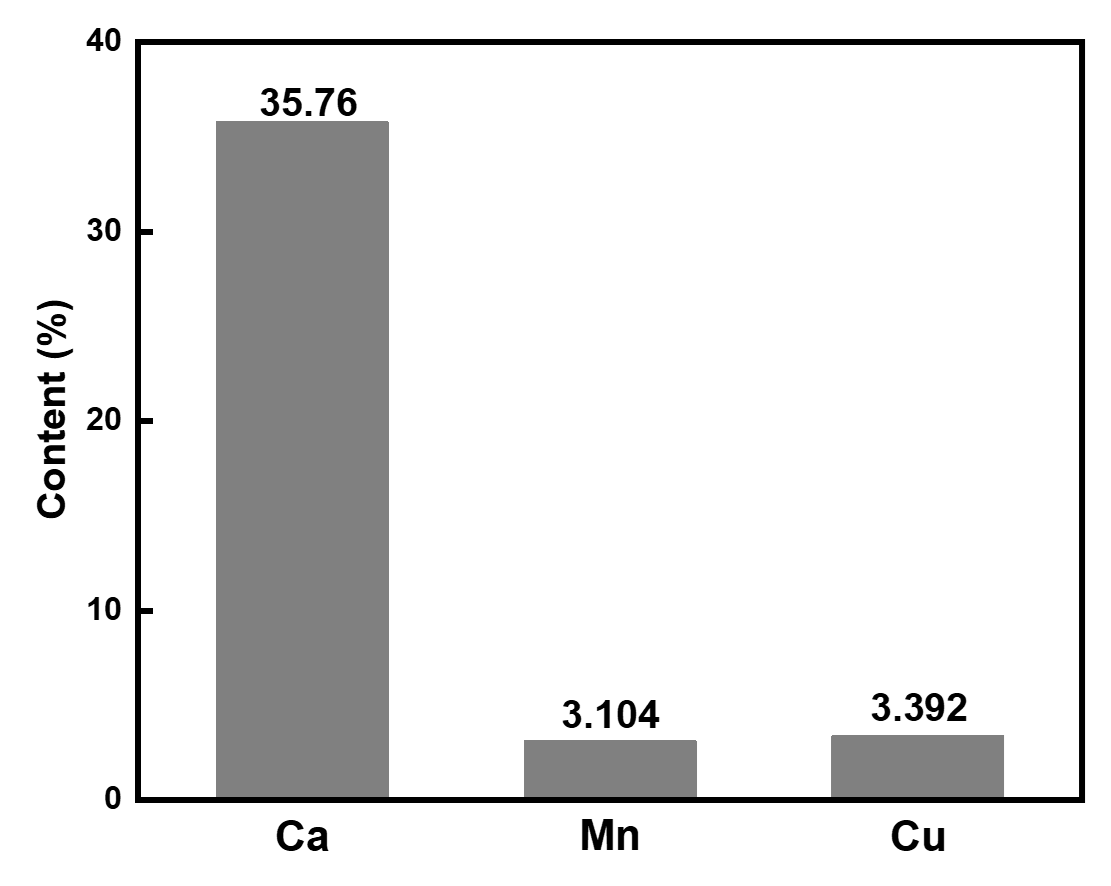


**Figure S2.** Elemental content analysis of CaCO_3_/Mn/Cu@lip-Apt.


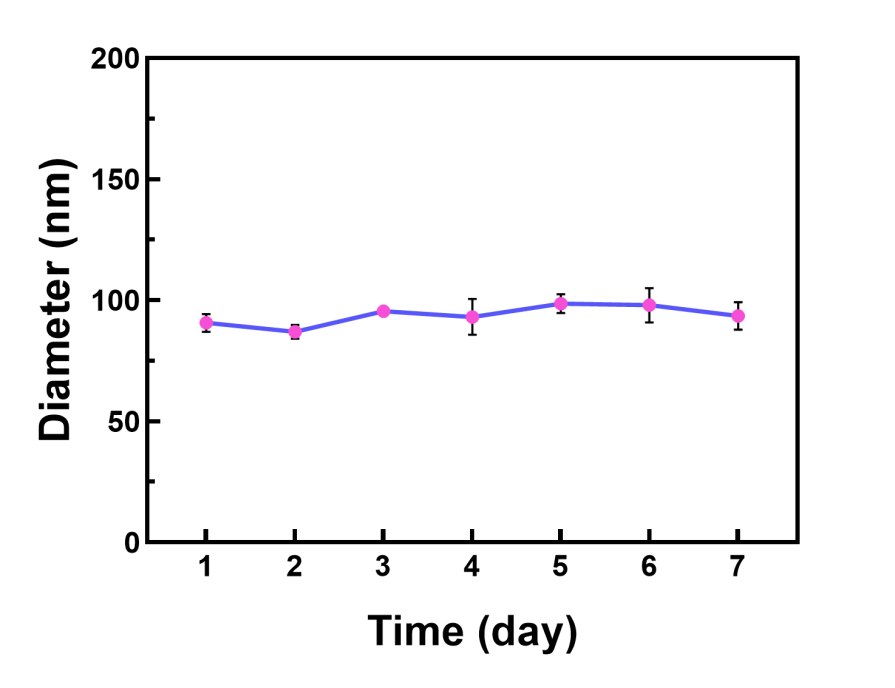


**Figure S3.** The diameters of CaCO_3_/Mn/Cu@lip-Apt in PBS for 7 days (n = 3).


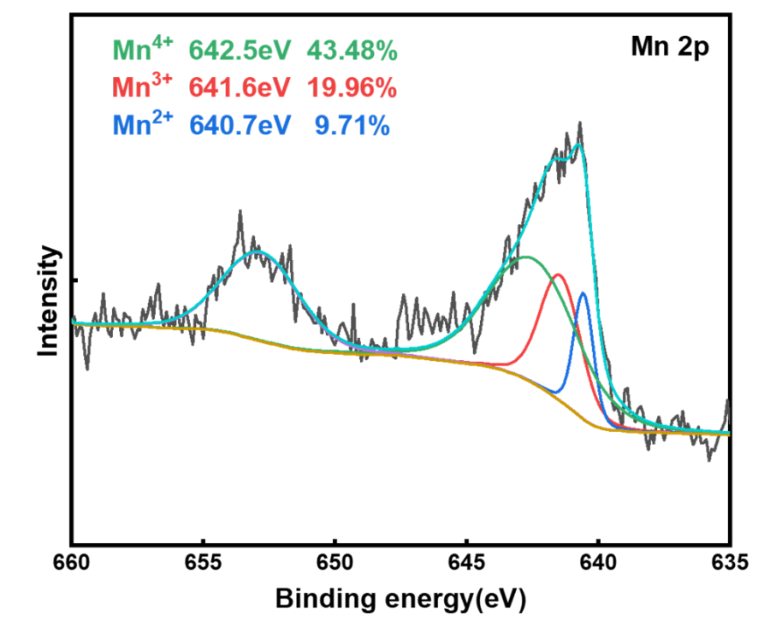


**Figure** **S4.** High-resolution Mn 2p XPS spectrum of CaCO_3_/Mn/Cu@lip-Apt.


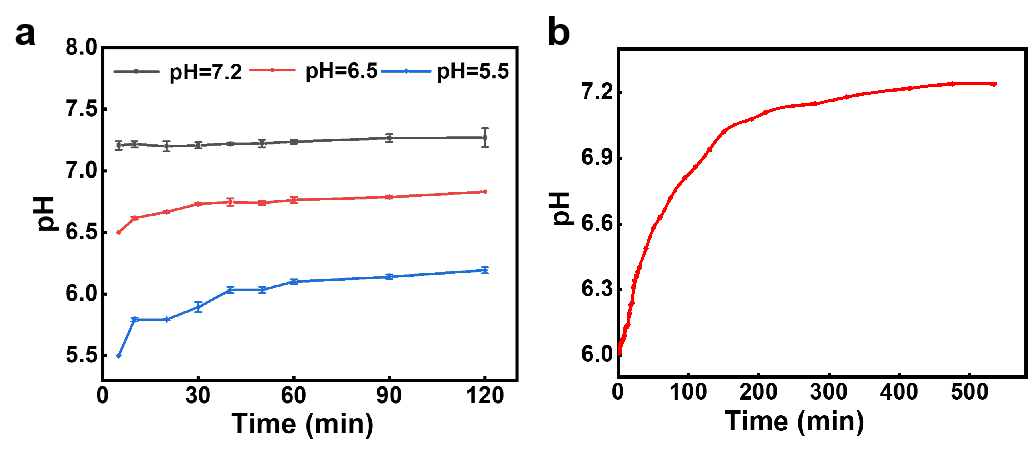


**Figure S5.** (a) After CaCO_3_/Mn/Cu@lip-Apt was incubated in HCl solutions at different pH, the pH value of the solution changed over time (n=3). (b) pH curve of CaCO_3_/Mn/Cu@lip-Apt after a long incubation at pH 6.5.


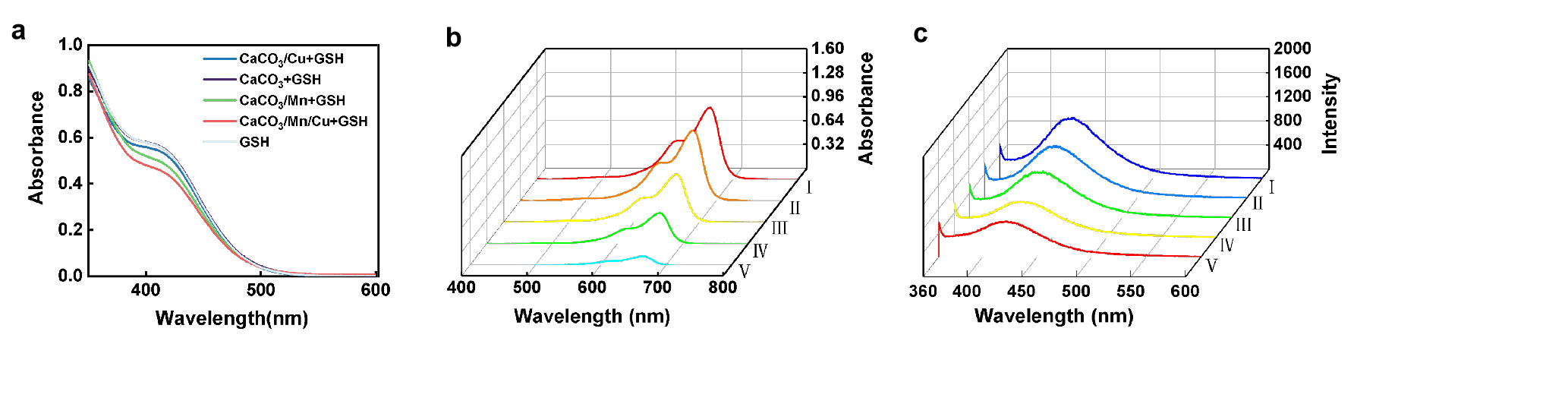


**Figure S6.** (a) The absorption curve of the solution after the reaction of various NPs with GSH. (b) Absorbance spectra of MB in various solutions. (Ⅰ: MB+H_2_O_2_+GSH; Ⅱ: MB+CaCO_3_+H_2_O_2_+GSH; Ⅲ: MB+CaCO_3_/Mn+H_2_O_2_+GSH; Ⅳ: MB+CaCO_3_/Cu+H_2_O_2_+GSH; Ⅴ: MB+CaCO_3_/Mn/Cu+H_2_O_2_+GSH.) (c) Fluorescence spectra of •OH generated by different NPs with TPA. (Ⅰ: TPA+H_2_O_2_+CaCO_3_/Mn/Cu+GSH; Ⅱ: TPA+H_2_O_2_+CaCO_3_/Cu+GSH; Ⅲ: TPA+H_2_O_2_+CaCO_3_/Mn+GSH; Ⅳ: TPA+H_2_O_2_+CaCO_3_+GSH, Ⅴ: TPA+H_2_O_2_+GSH.)


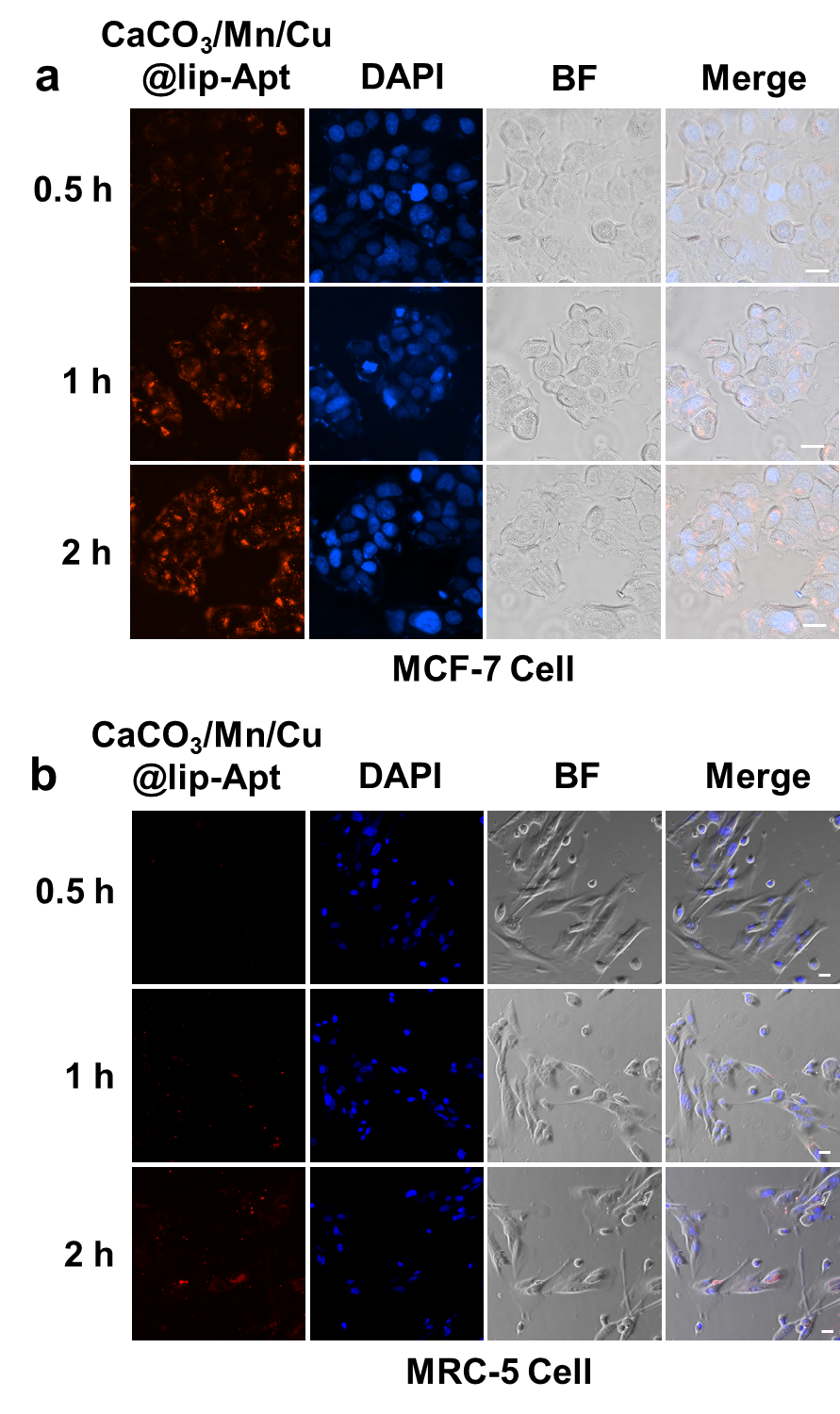


**Figure S7.** Fluorescence images of cells incubated with CaCO_3_/Mn/Cu@lip-Apt for 0.5, 1, and 2 h, respectively. (a) MCF-7 cells and (b) MRC-5 cells. Scale bar: 20 μm.


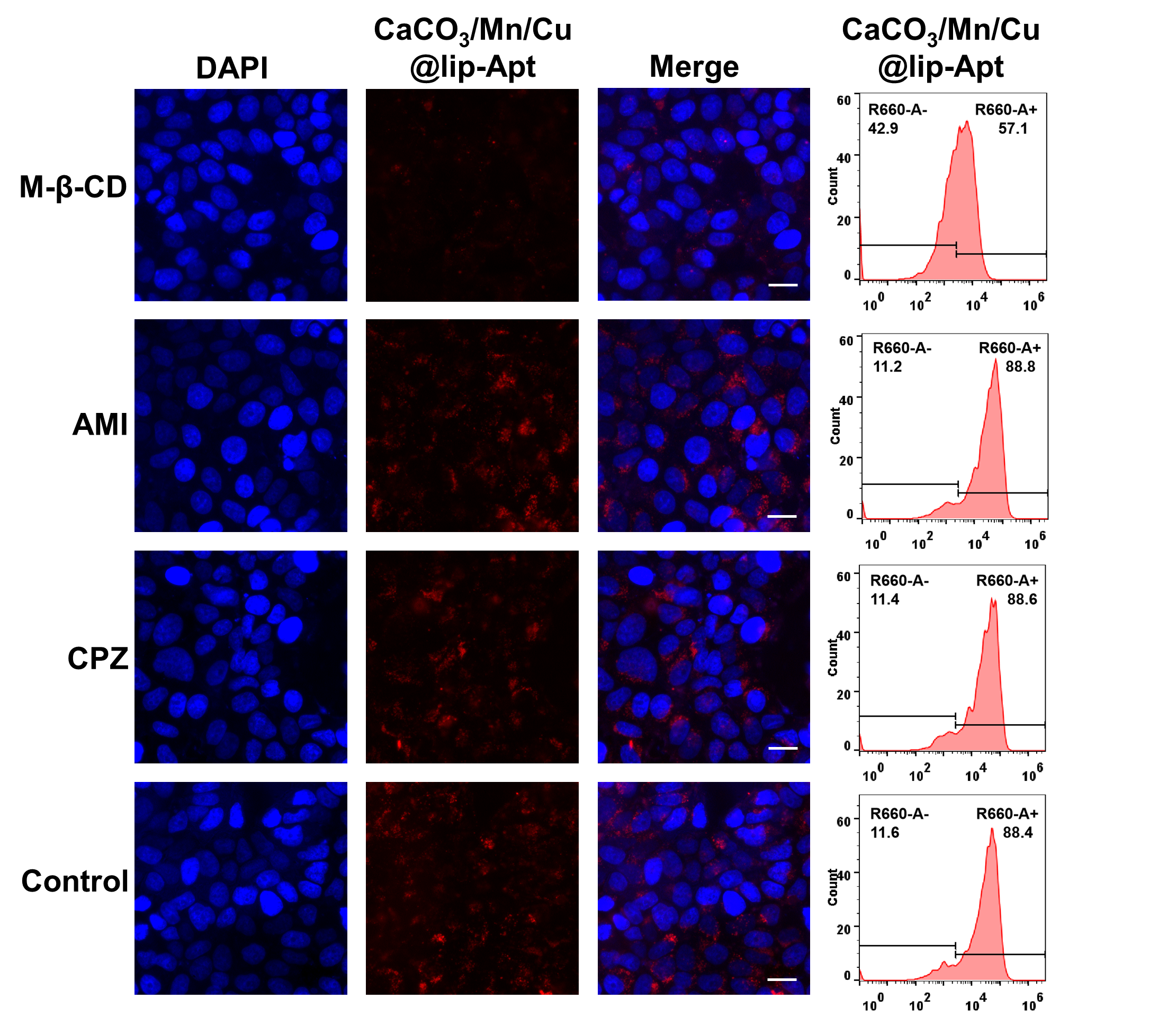



**Figure S8.** Fluorescence image and flow cytometry assay of cells incubating with CPZ, AMI, and M-β-CD for 1 h. The control group was not treated with endocytosis inhibitors. Cells in each group were incubated with CaCO_3_/Mn/Cu@lip-Apt for 2 h. Scale bar: 20 μm.


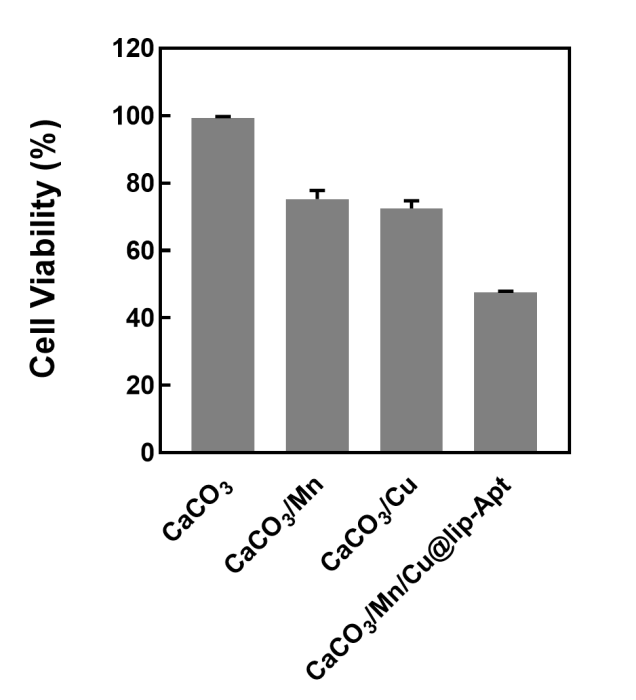


**Figure S9.** Cell viability was assessed after incubating cells with CaCO_3_, CaCO_3_/Mn, CaCO_3_/Cu, and CaCO_3_/Mn/Cu@lip-Apt (n=3).

**
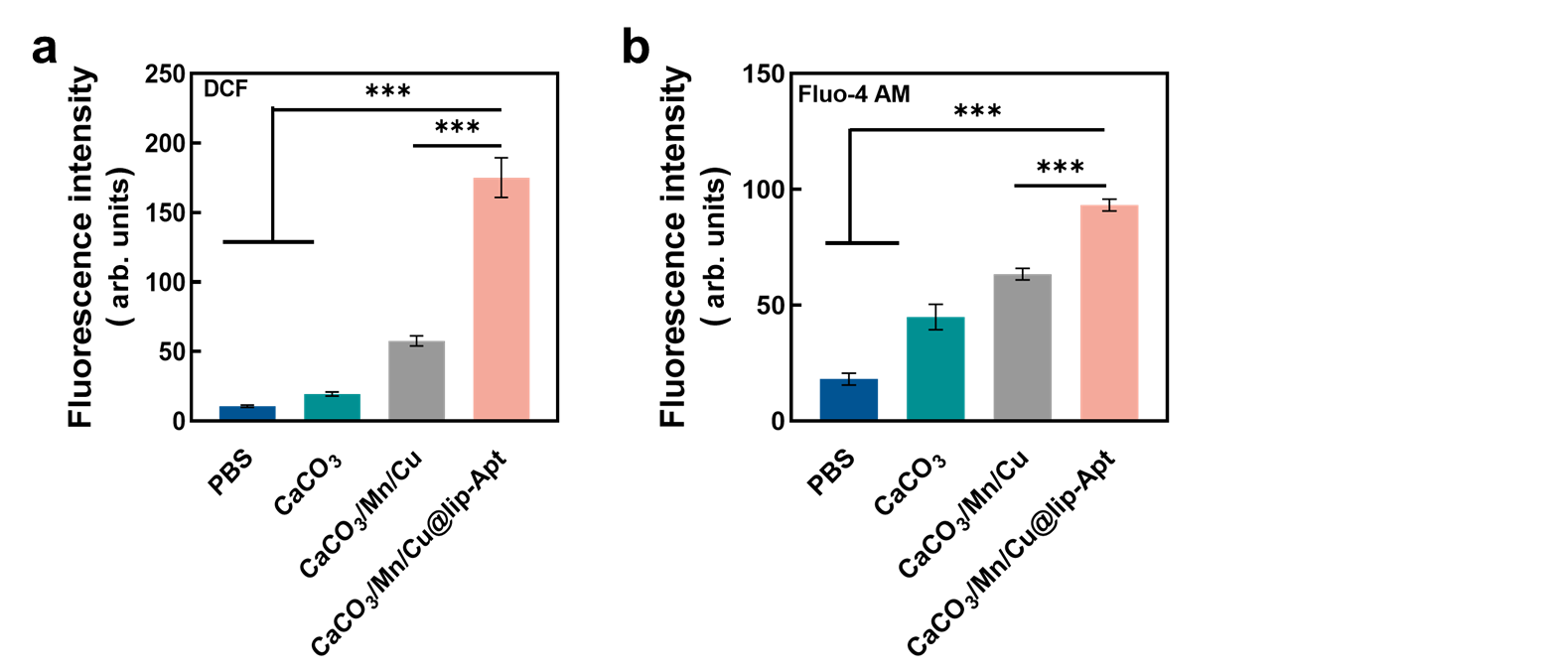
**

**Figure S10.** (a) Quantification of fluorescence for DCF related to Figure 5a. (b) Quantification of fluorescence for Flou-4 AM related to Figure 5b. Data are represented as mean ± SD. p values were calculated via one-way ANOVA test in Figure S11a-b. ***p < 0.001 (n=3).


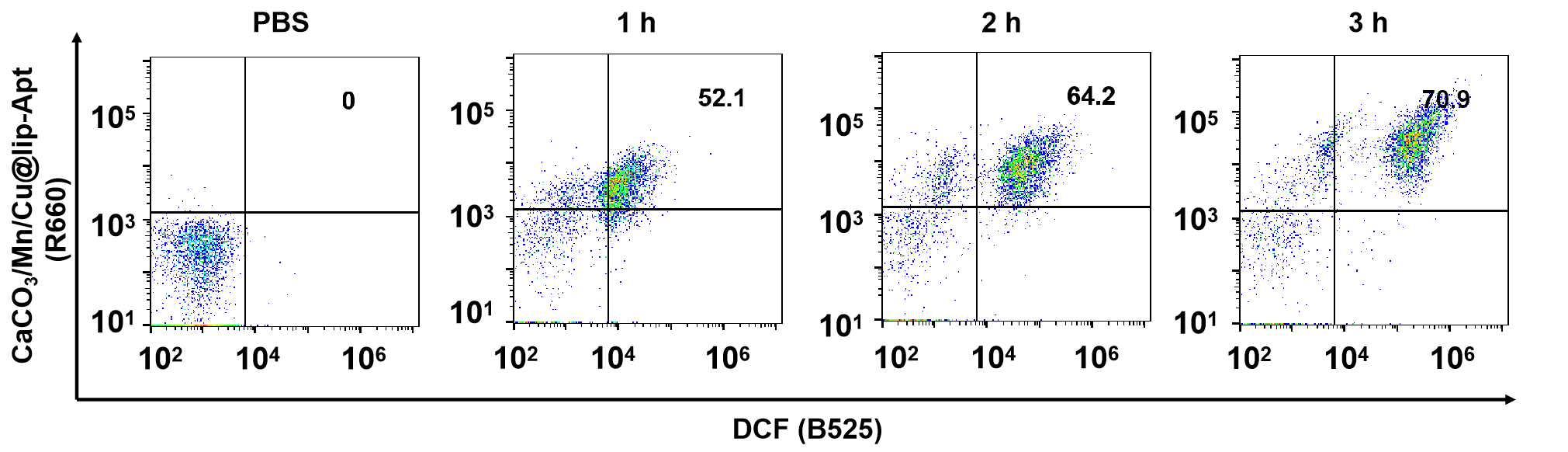


**Figure S11.** Flow cytometry was used to detect the DCF content after incubation of CaCO_3_/Mn/Cu@lip-Apt and MCF-7 cells for different times.


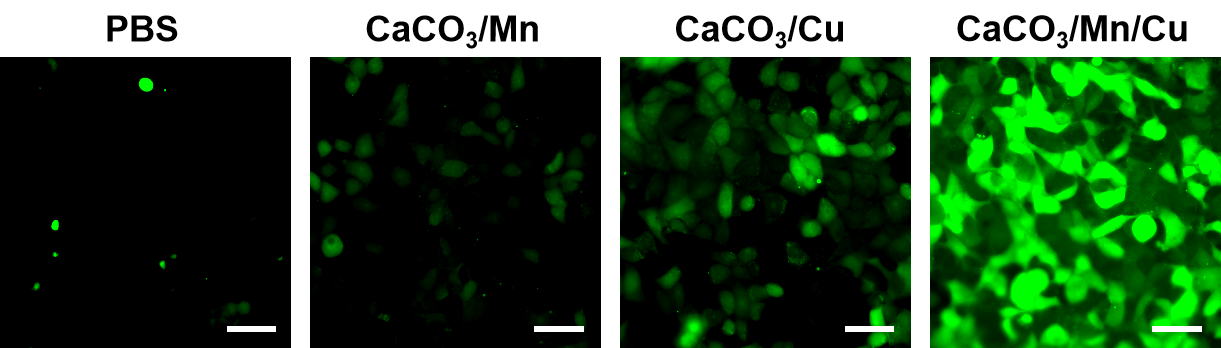


**Figure S12.** Fluorescence images of MCF-7 cells after incubation with different materials, using DCFH-DA to evaluate ROS production. Scale bar: 50 μm.


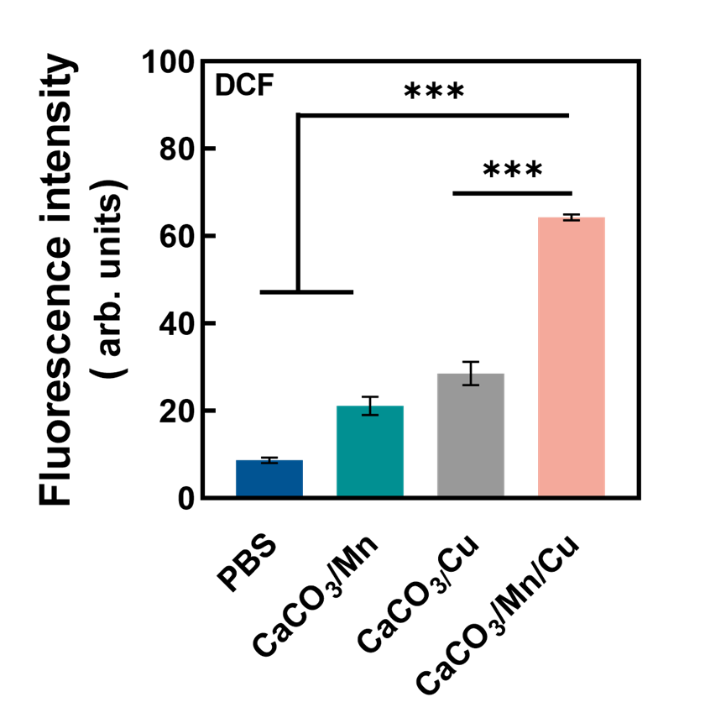


**Figure S13.** Quantification of fluorescence for DCF. Related to Figure S12. Data are represented as mean ± SD. p values were calculated via one-way ANOVA test. ***p < 0.001 (n=3).


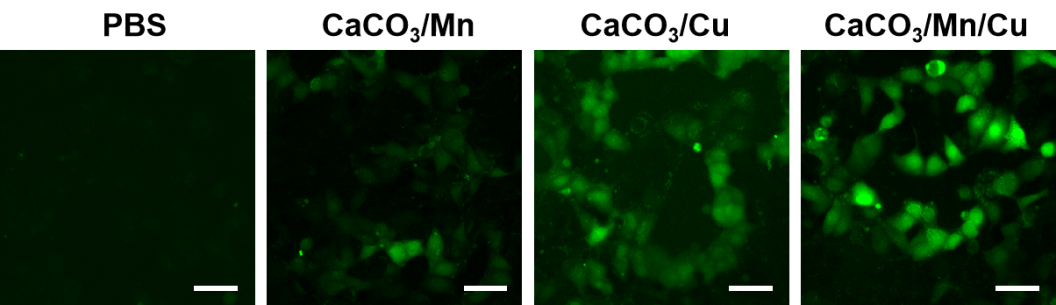


**Figure S14.** Fluorescence image of Fluo-4 AM after MCF-7 cells incubated with CaCO_3_/Mn, CaCO_3_ /Cu and CaCO_3_/Mn/Cu. Scale bar: 50 μm.


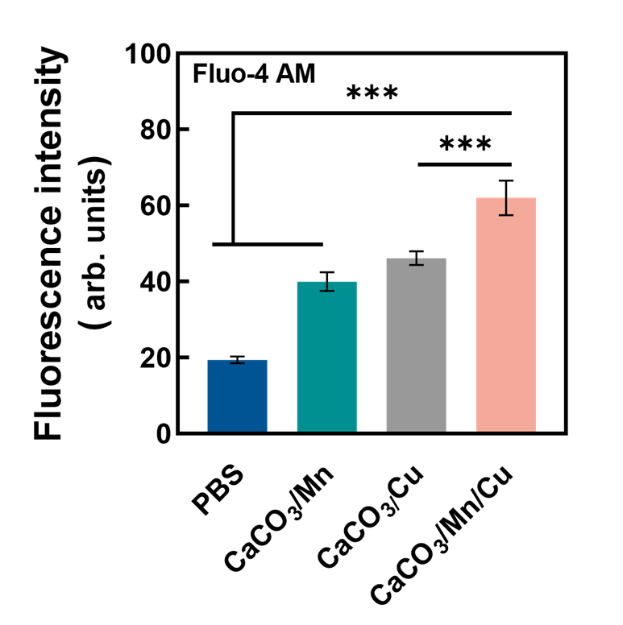


**Figure S15.** Quantification of fluorescence of Fluo-4 AM in Figure S14. Data are represented as mean ± SD. p values were calculated via one-way ANOVA test. ***p < 0.001 (n=3).


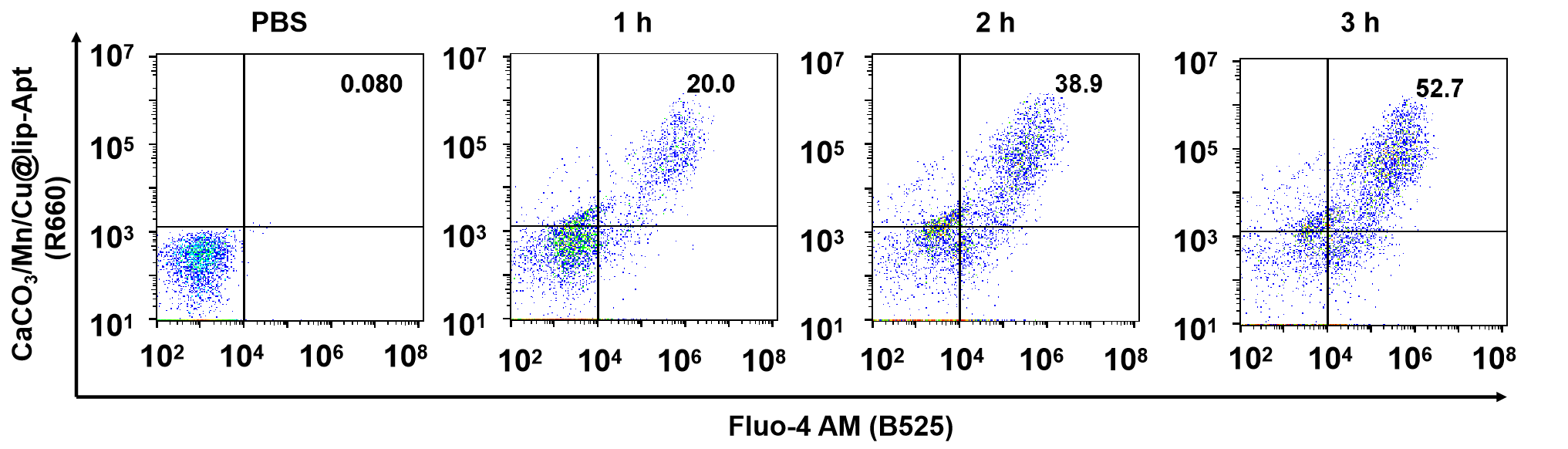


**Figure S16.** The Ca^2+^ concentration was detected by flow cytometry after coincubation with CaCO_3_/Mn/Cu@lip-Apt and MCF-7 cells at different time (Ca^2+^ was detected by Fluo-4AM).


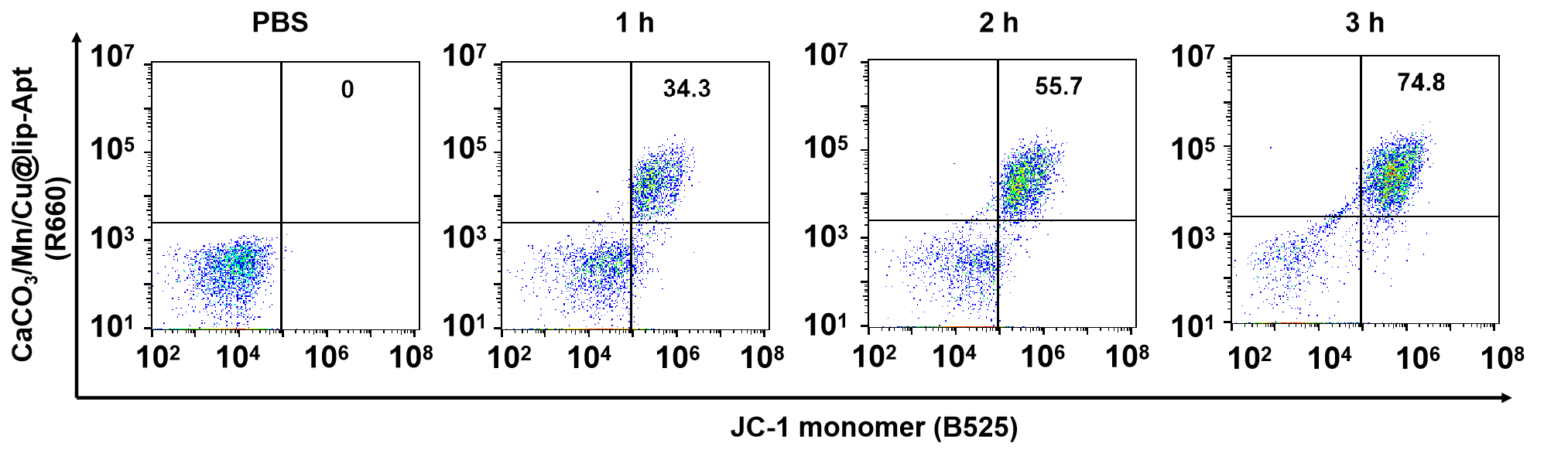


**Figure S17.** Flow cytometry was used to monitor changes in JC-1 monomer after incubation of CaCO_3_/Mn/Cu@lip-Apt and MCF-7 cells for different times.


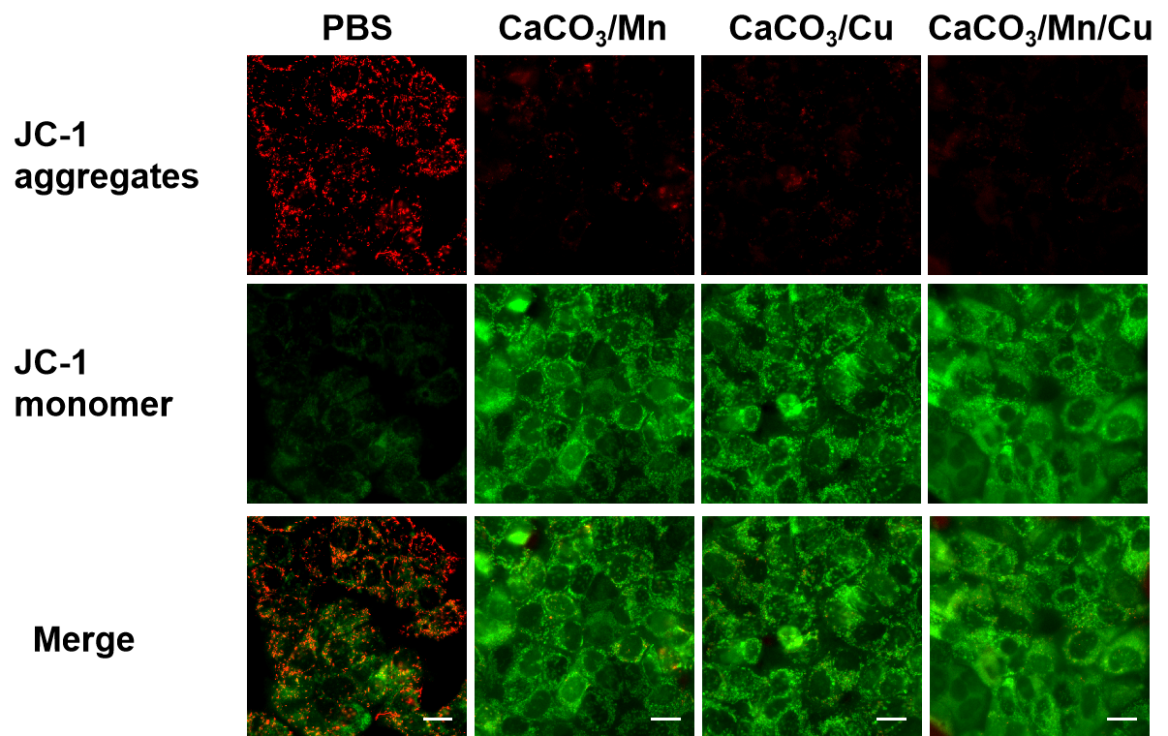


**Figure S18.** Fluorescence image of MCF-7 cells (*vs* JC-1) after incubation with CaCO_3_/Mn, CaCO_3_/Cu and CaCO_3_/Mn/Cu. Scale bar: 20 μm.


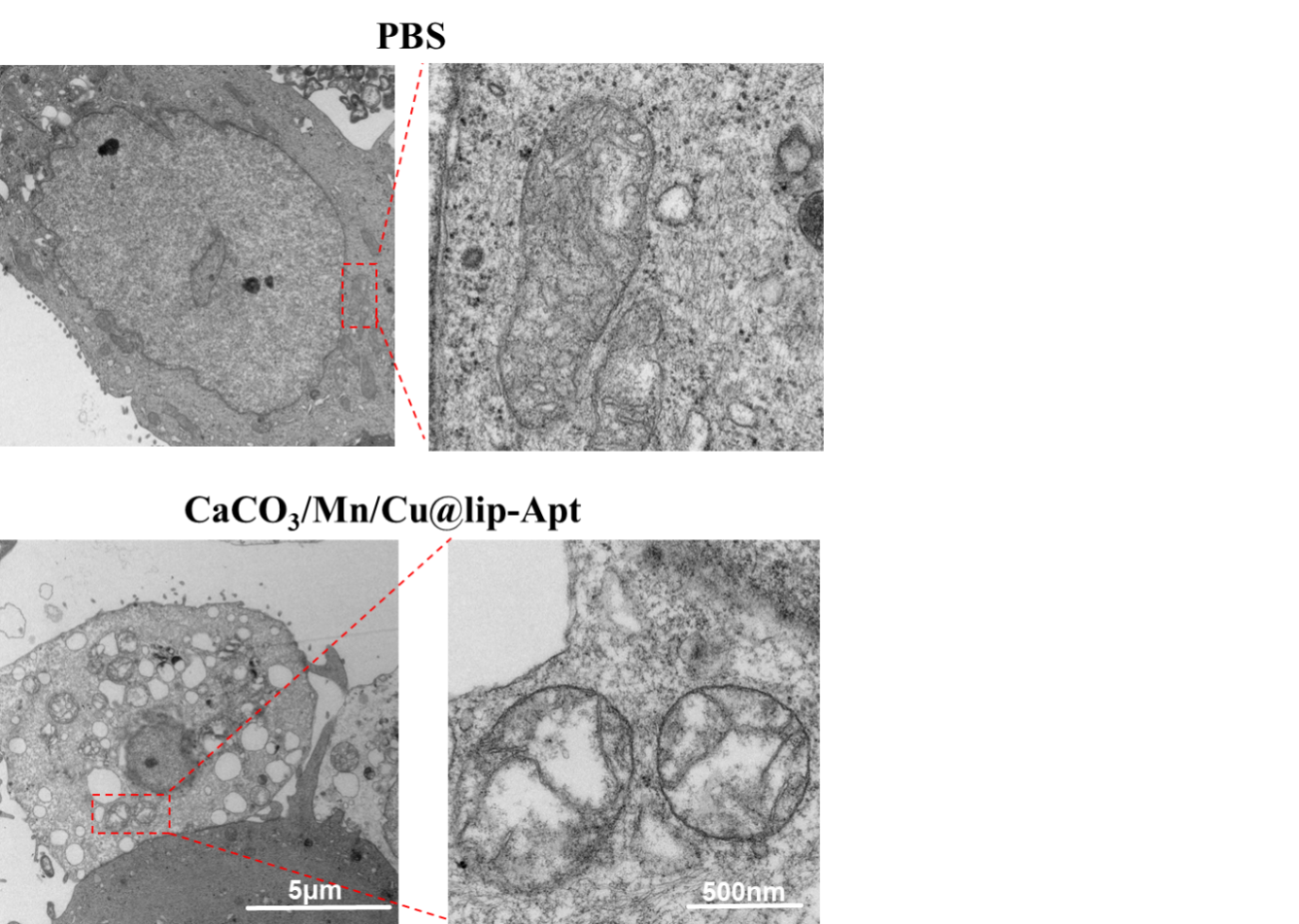


**Figure S19.** Representative Bio-TEM images of MCF-7 cells incubated with PBS and CaCO_3_/Mn/Cu@lip-Apt. The red box marked the location of mitochondria.


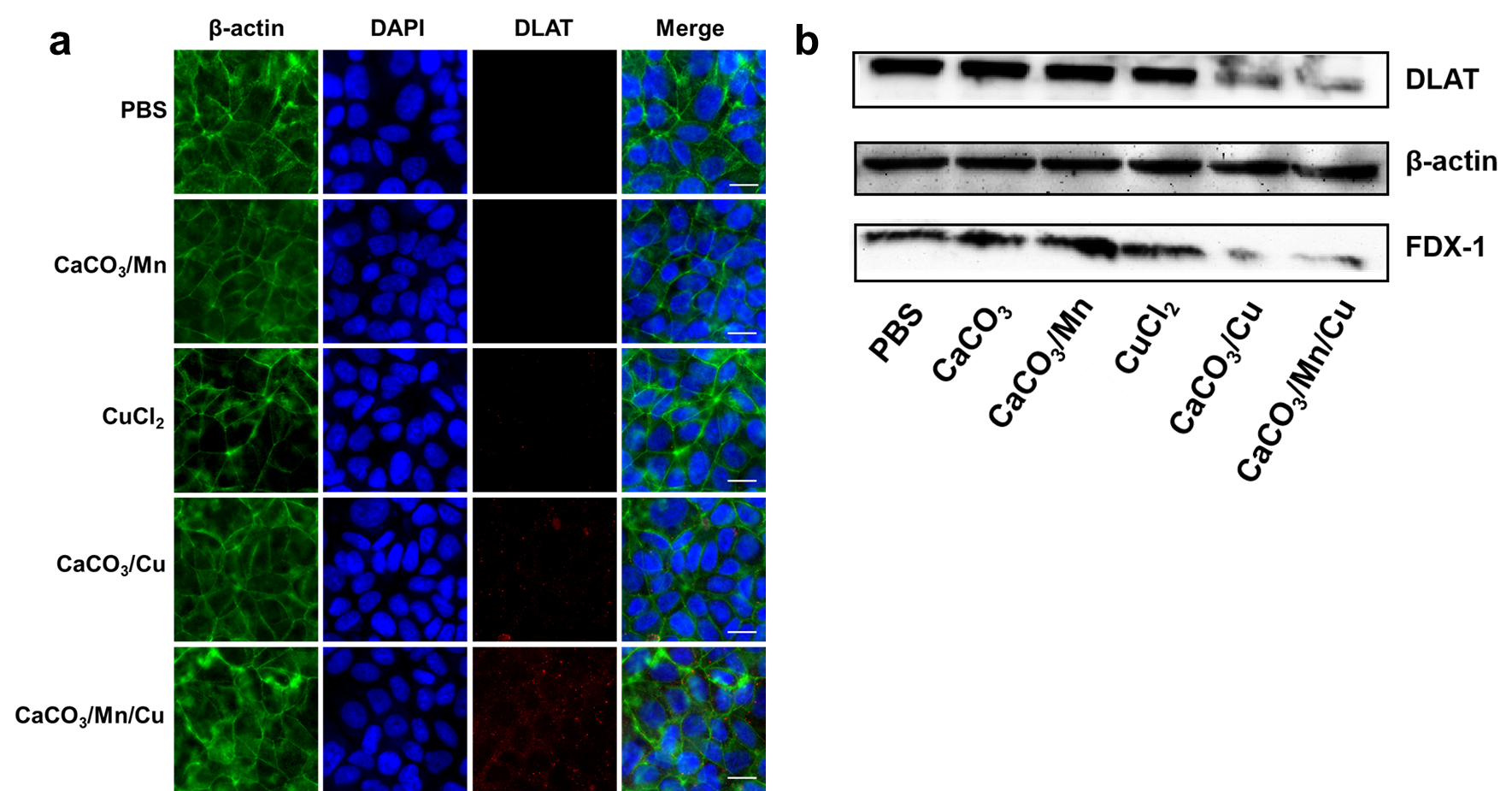


**Figure S20.** (a) Immunofluorescence images of cells in different groups. Scale bar: 20 μm. (b) Western blot analysis was performed on the protein expression of cuproptosis related proteins (FDX-1 and DLAT) in MCF-7 cells.


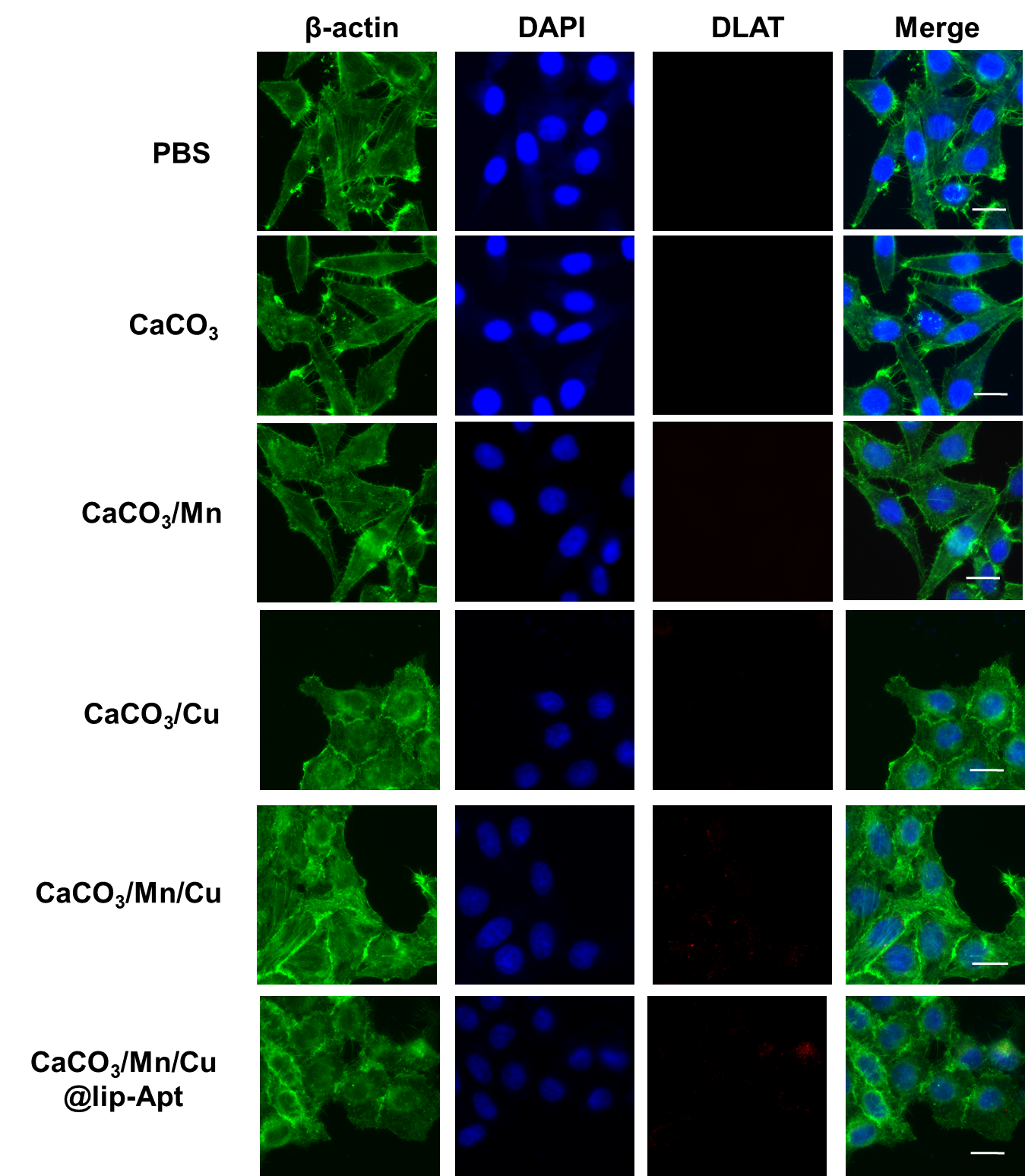


**Figure S21.** DLAT immunofluorescence images of HepG2 cells in different groups. The CaCO_3_/Mn/Cu@lip-Apt used here did not contain DiD. Scale bar = 20 μm.


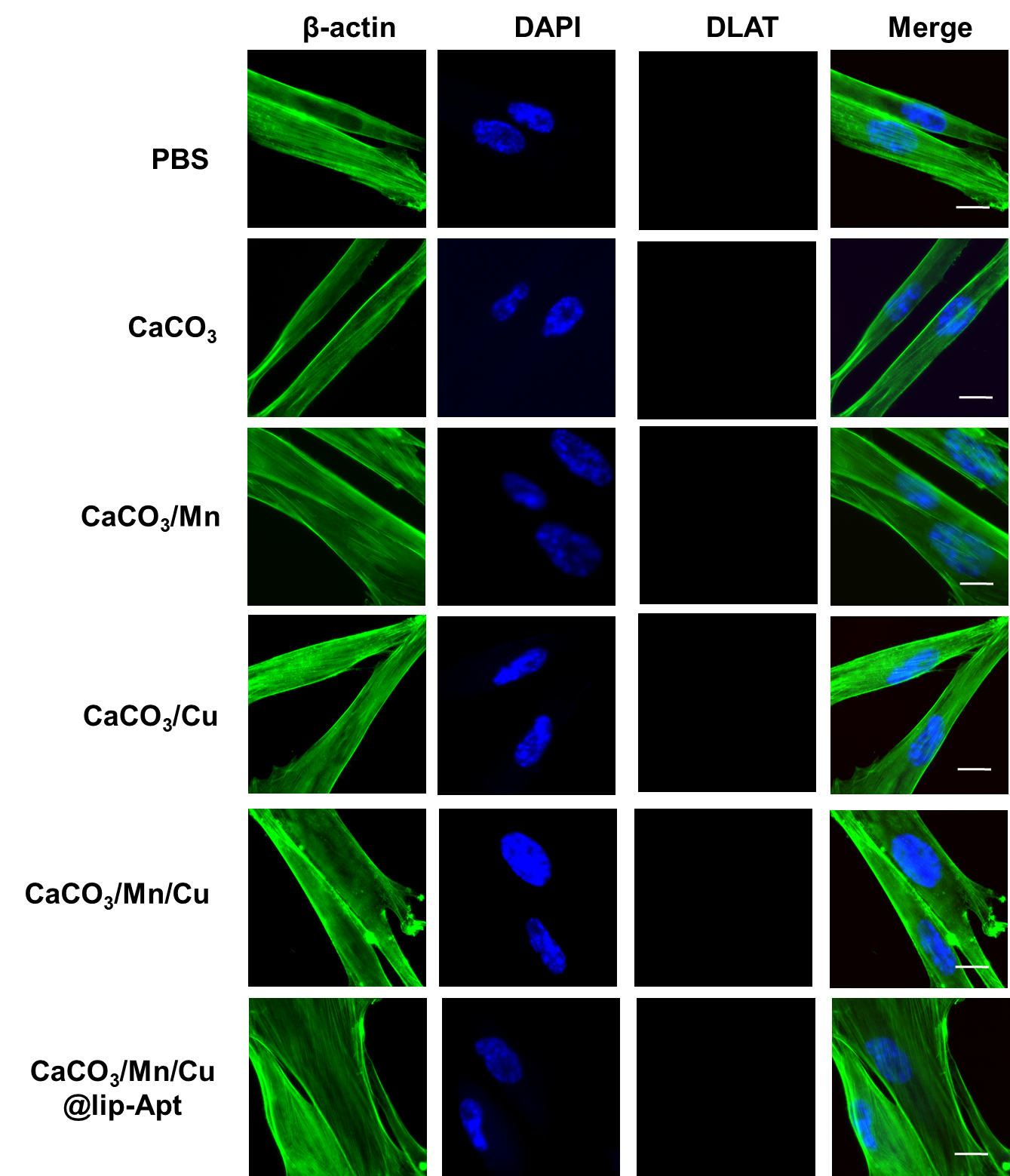


**Figure S22.** DLAT immunofluorescence images of MRC-5 cells in different groups. The CaCO_3_/Mn/Cu@lip-Apt used here did not contain DiD. Scale bar = 20 μm.

**
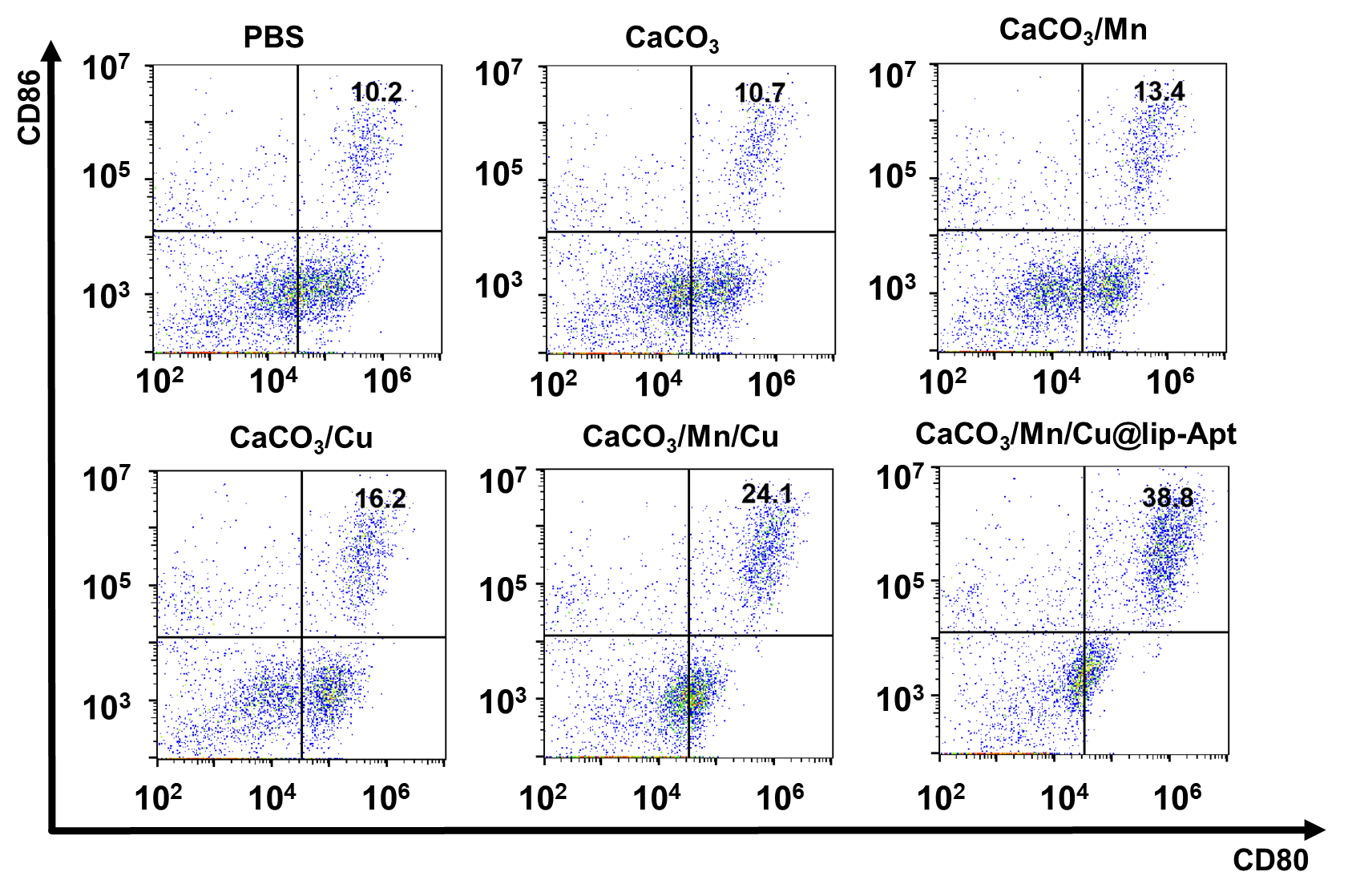
**

**Figure S23.** Flow cytometry analysis of BMDCs activation, characterized by CD86 and CD80 expression. The CaCO_3_/Mn/Cu@lip-Apt used here did not contain DiD.


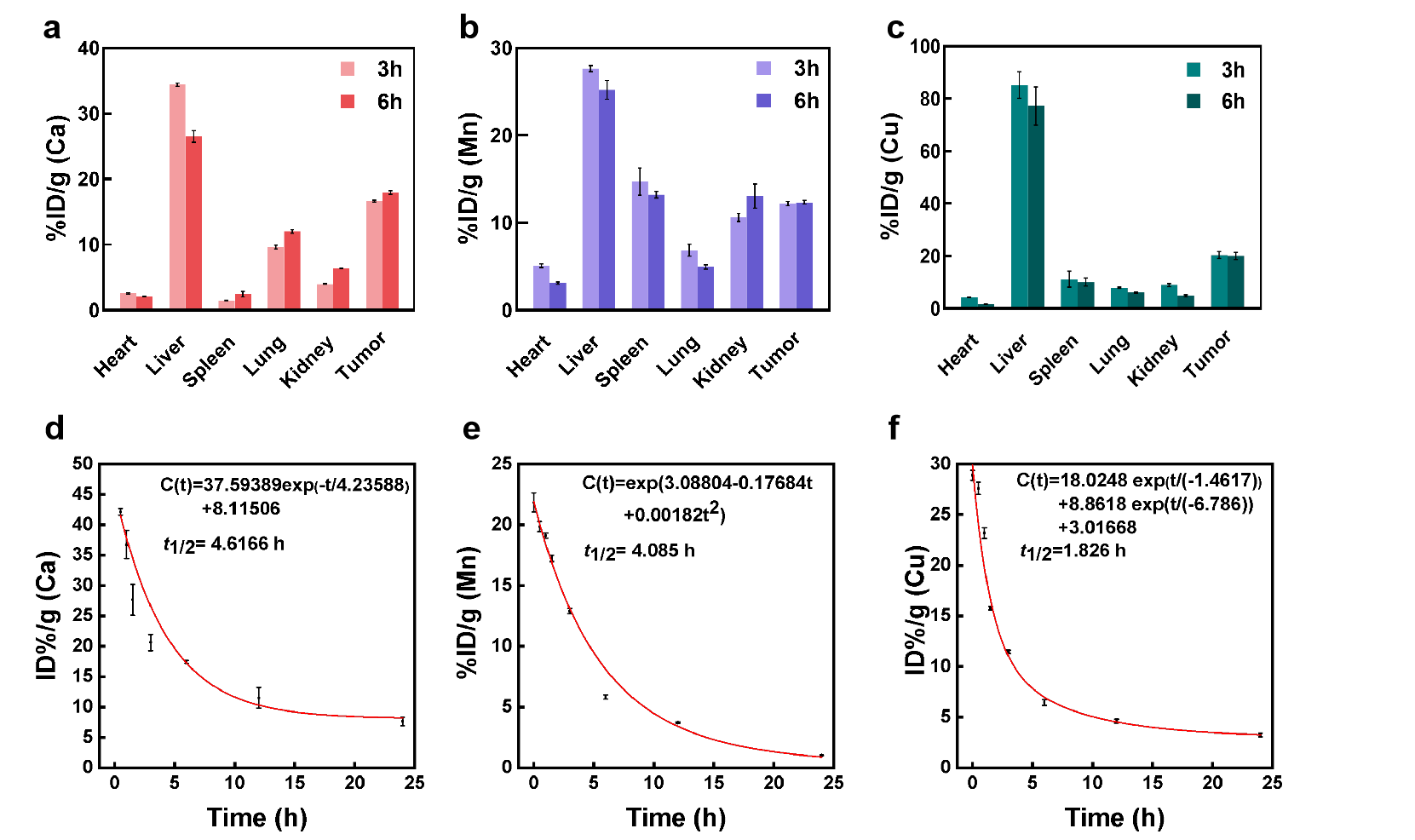


**Figure S24.** (a-c) Biodistribution of Ca(a), Mn(b) and Cu(c) in major organs and tumors after tail vein injection of CaCO_3_/Mn/Cu@lip-Apt at different time intervals (% injectable dose (ID) of metal elements per gram of tissue, n = 3 biologically independent samples) (d-f) Blood circulation of Ca(d), Mn(e) and Cu(f) in mice after intravenous injection of CaCO_3_/Mn/Cu@lip-Apt.


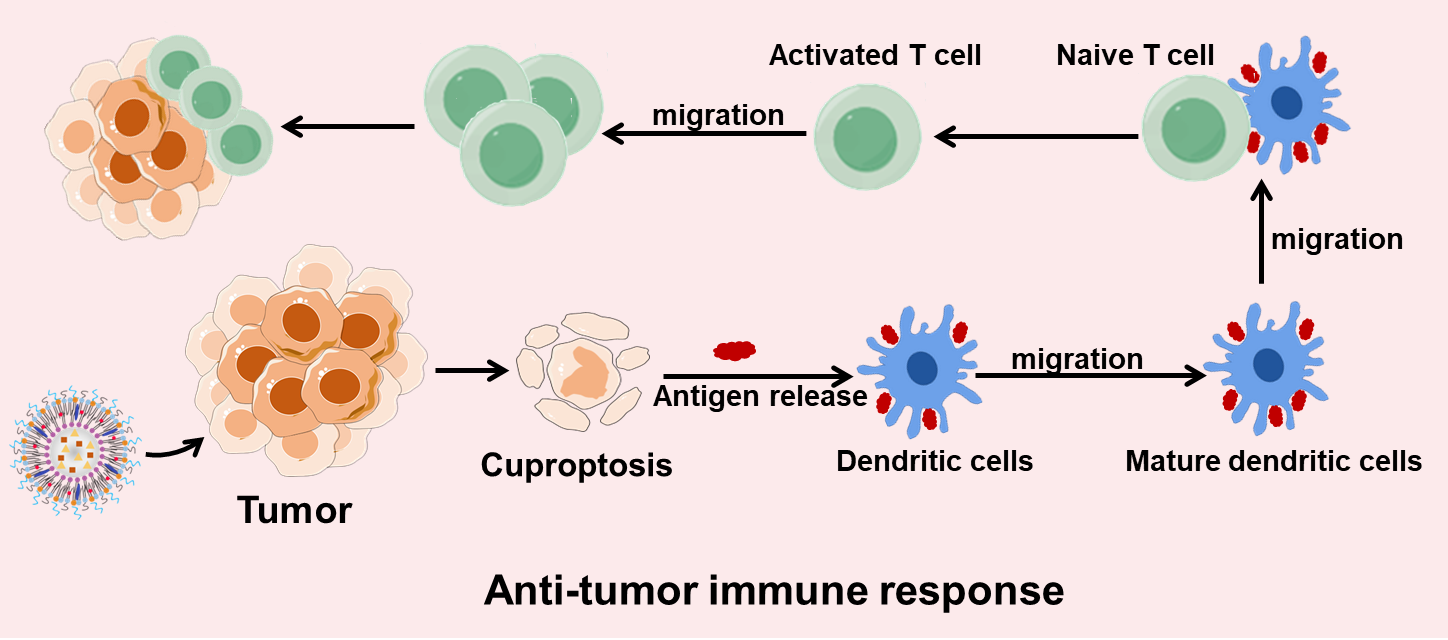


**Figure S25.** The schematic diagram of cuprotosis induce-ICD.


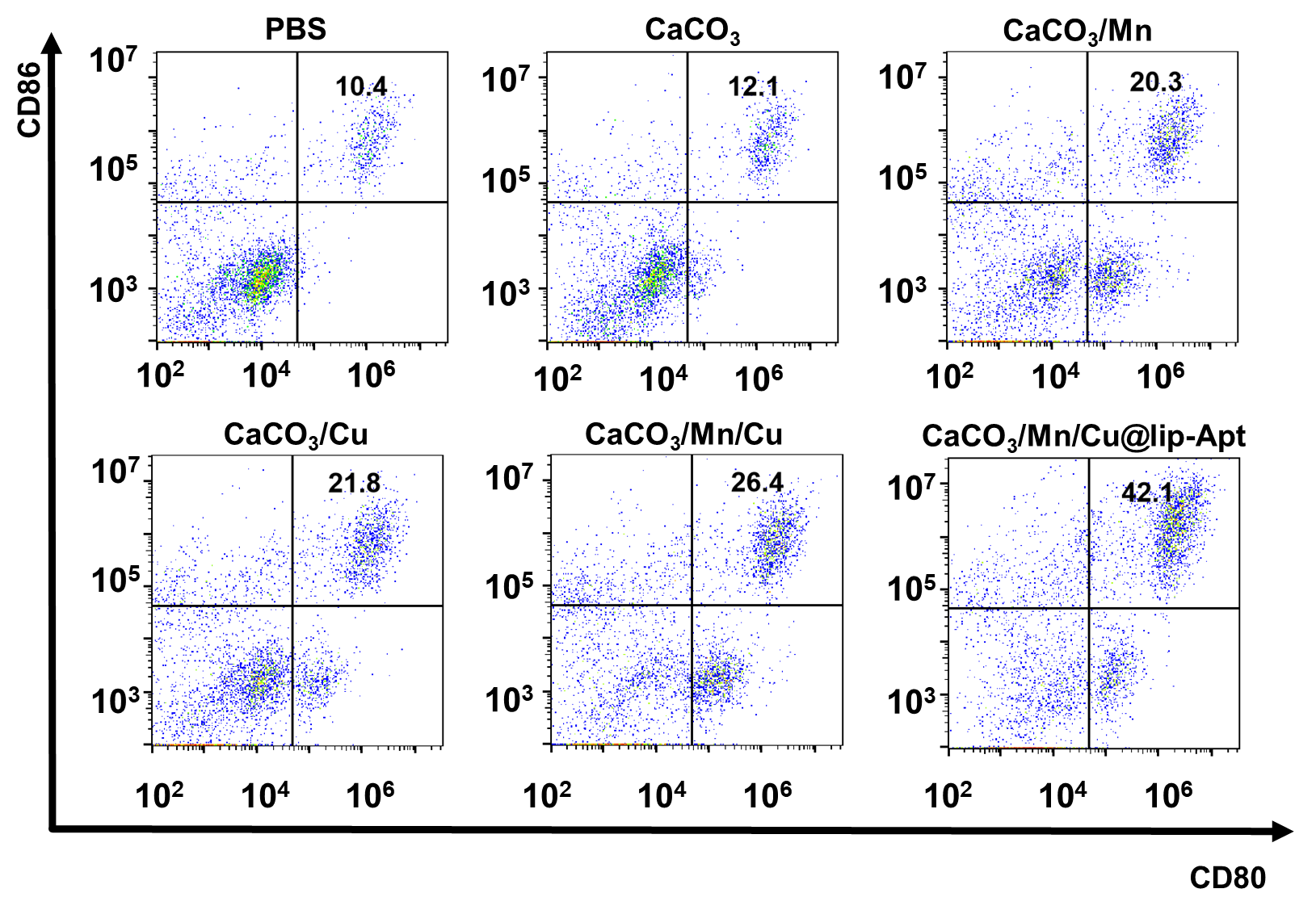


**Figure S26.** Representative flow cytometry analysis profiles of matured DC (CD80, CD86) populations in the tumors with various treatments. The CaCO_3_/Mn/Cu@lip-Apt used here did not contain DiD.


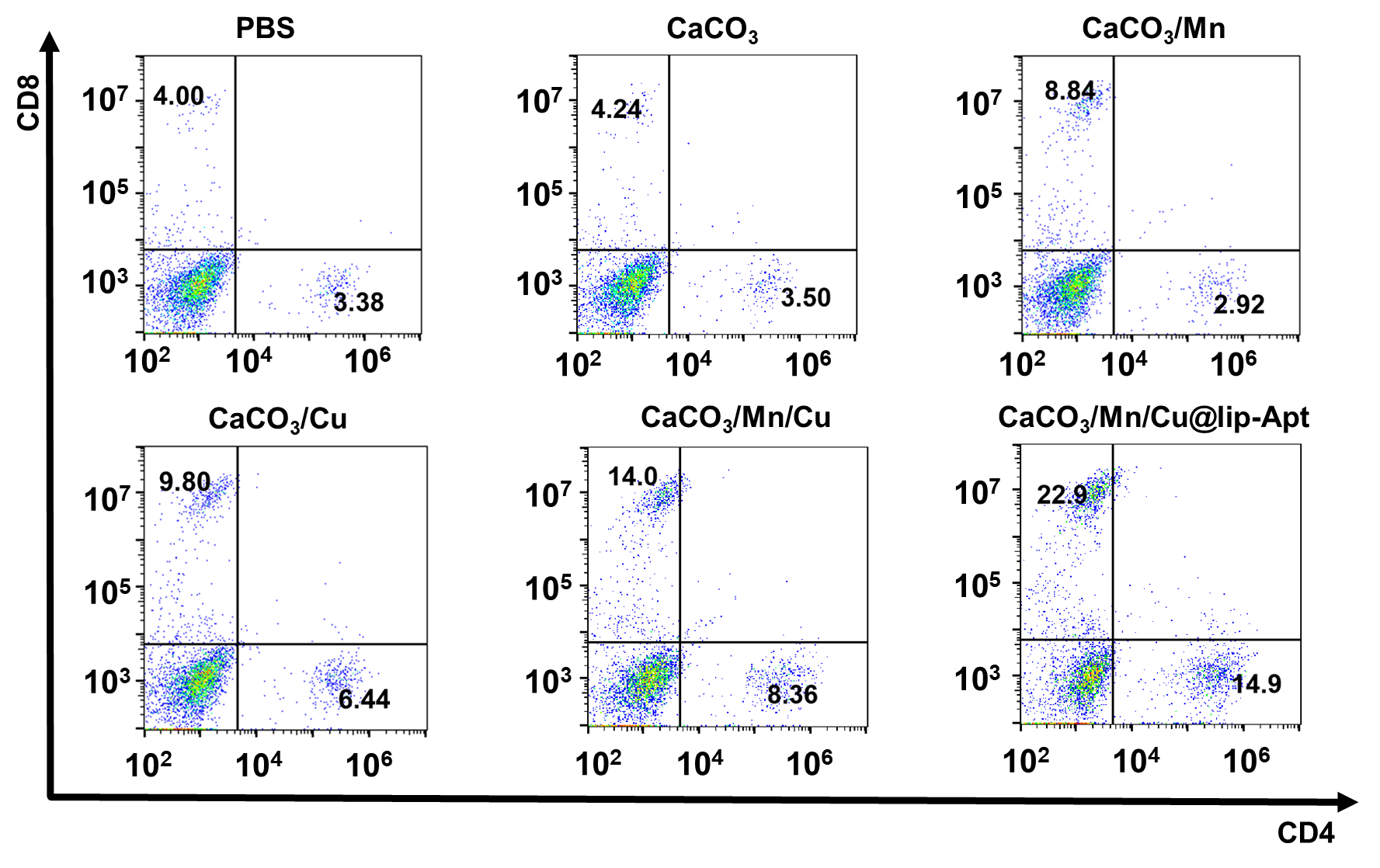


**Figure S27.** Analysis of CD4^+^, CD8^+^ T cells in tumors of each group using flow cytometry. The CaCO_3_/Mn/Cu@lip-Apt used here did not contain DiD.


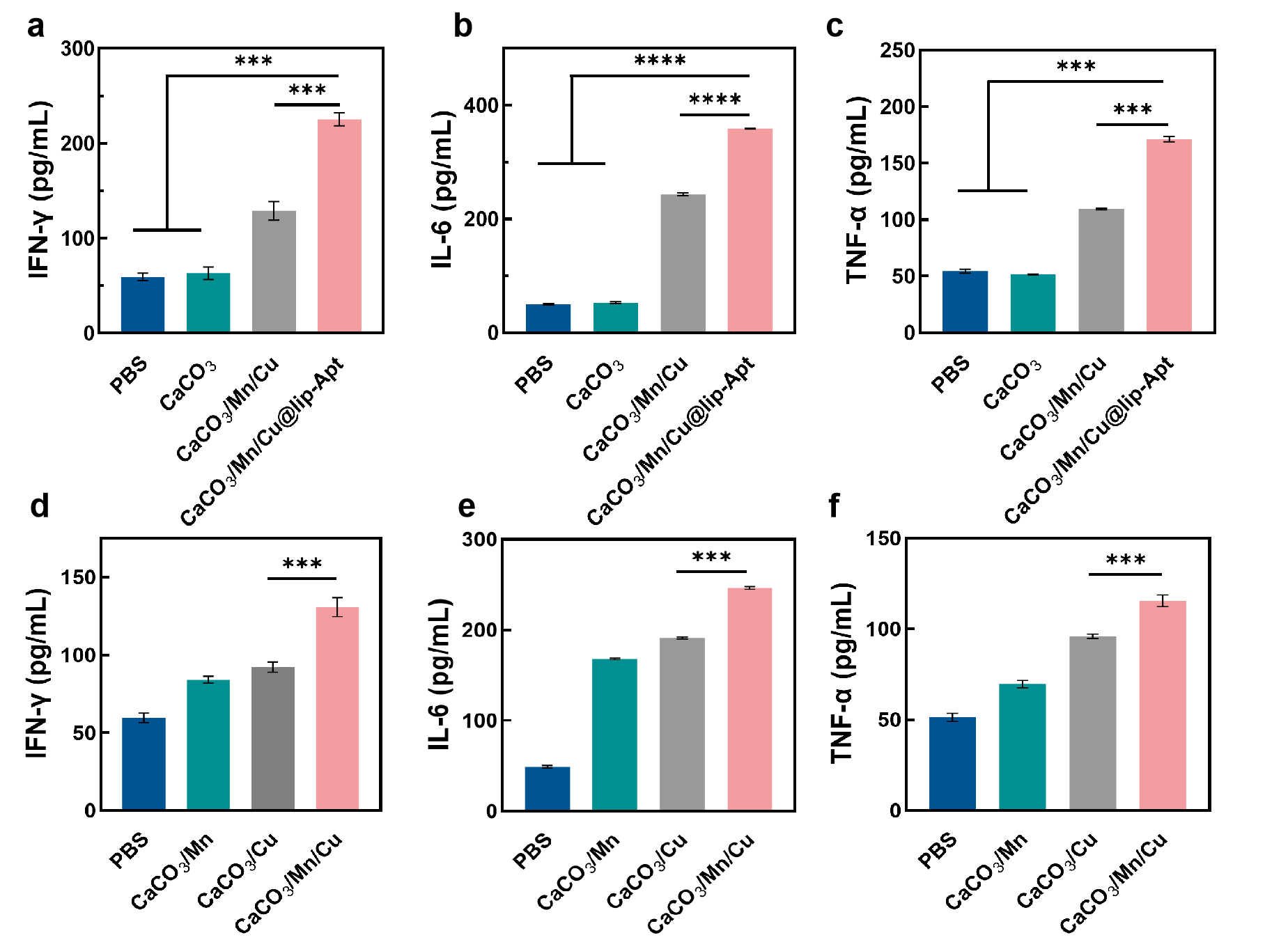


**Figure S28.** Levels of IFN-γ, IL-6, and TNF-α in the serum of mice treated with different materials (a-f were the data obtained from different batches of experiments). Data are represented as mean ± SD. p values were calculated via one-way ANOVA test in a-f. ***p < 0.001, ****p < 0.0001, (n=3). The above experiments were performed using six separate 96-well plates.


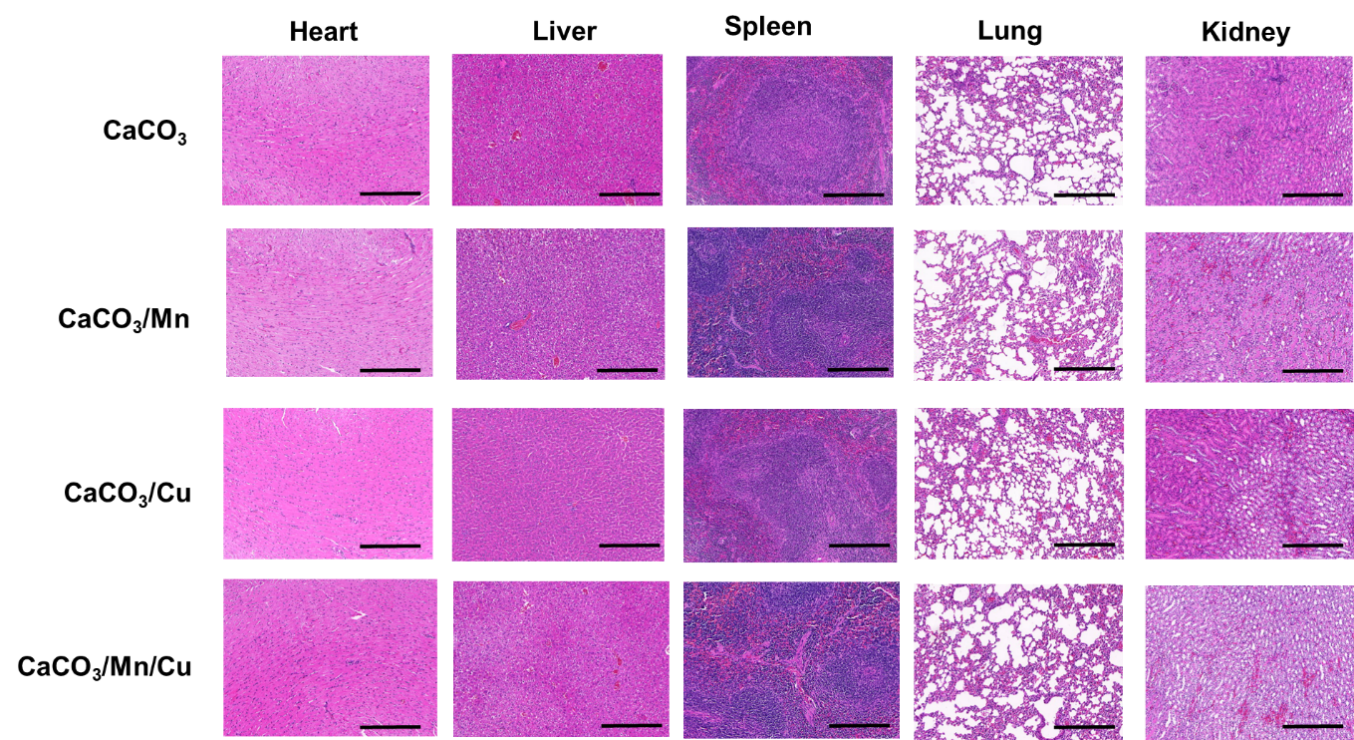


**Figure S29.** Histological analysis of the organ using a hematoxylin and eosin stain after various treatment. Scale bar: 200 μm.


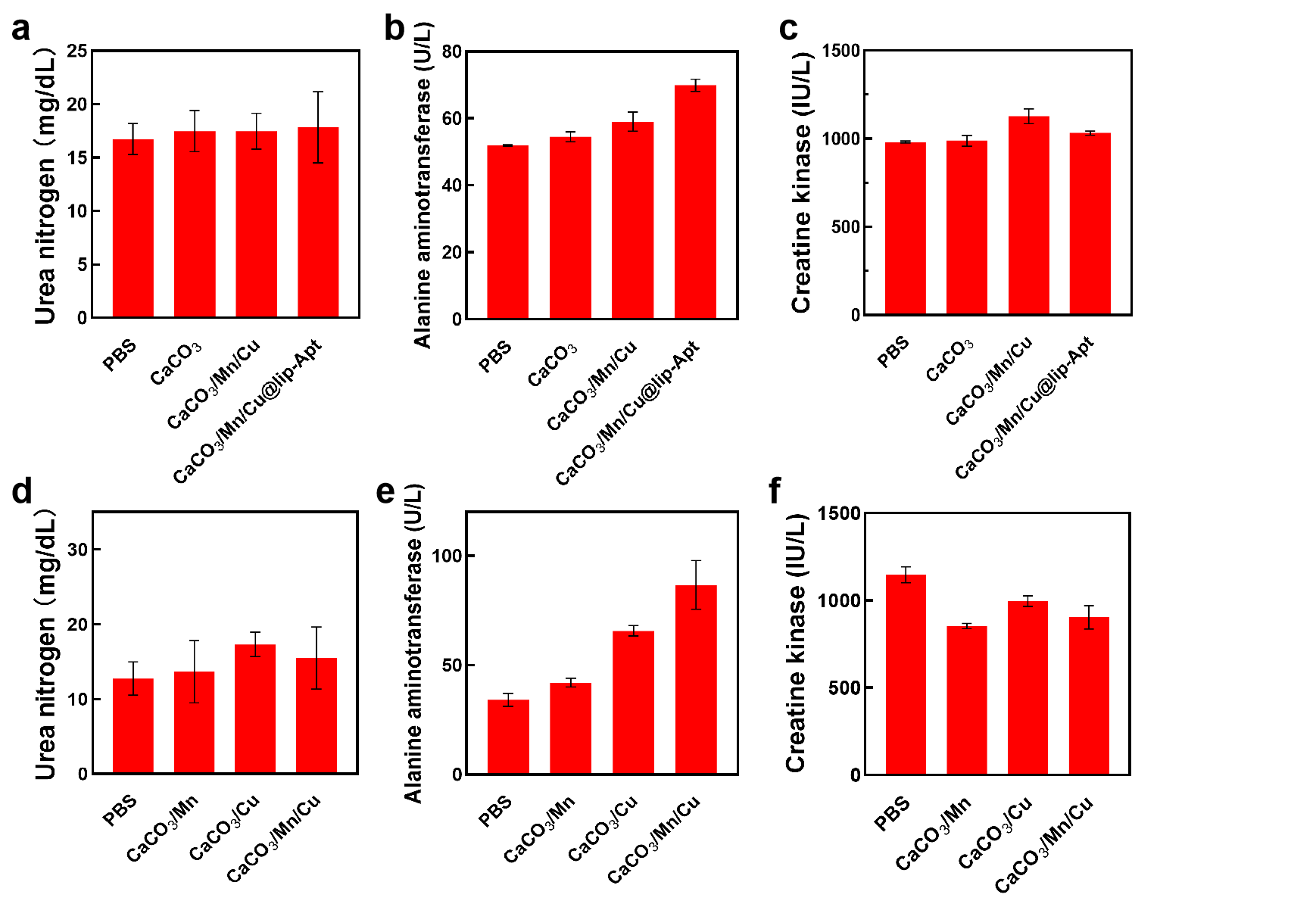


**Figure S30.** Serum biochemistry analysis of urea nitrogen, alanine aminotransferase, creatinine kinase of mice for different group (n=3). a-f were the data obtained from different batches of experiments.

References

[1] T. A. Mir, J. H. Yoon, N. G. Gurudatt, M. S. Won, & Y. B. Shim, *Biosens. Bioelectron.* **2015**, 74, 594-600.

[2] P. Wu, Y. Gao, H. Zhang, C. Cai, *Anal. Chem.*, **2012**, 84, 7692-7699.

[3] H. Zu, Y. Wu, H. Meng, X. Cheng, Y. Wang, L. W. Zhang, Y. Wang, *ACS nano*, **2024,** 18, 23941-23957.

[4] C. He, N. Zhu, Y. Chen, Y. Zheng, S. Chen, Z. Wu, C. Yan, *Adv. Funct. Mater.*, **2024**, 2409966.
